# The REACT study: design of a randomized phase 3 trial to assess the efficacy and safety of clazosentan for preventing deterioration due to delayed cerebral ischemia after aneurysmal subarachnoid hemorrhage

**DOI:** 10.1186/s12883-022-03002-8

**Published:** 2022-12-20

**Authors:** Nicolas Bruder, Randall Higashida, Hugues Santin-Janin, Cécile Dubois, E. François Aldrich, Angelina Marr, Sébastien Roux, Stephan A. Mayer

**Affiliations:** 1 Department of Anesthesia and Critical Care, Hôpital de la Timone, Aix-Marseille Université, 264 rue St-Pierre, 13005 Marseille, France; 2 Department of Neuro Interventional Radiology, University of California San Francisco Medical Center, San Francisco, USA; 3 Biometry, Idorsia Pharmaceuticals Ltd, Allschwil, Switzerland; 4 Department of Neurosurgery, University of Maryland, Baltimore, USA; 5 Global Clinical Development, Idorsia Pharmaceuticals Ltd, Allschwil, Switzerland; 6 Neurocritical Care and Emergency Neurology Services, Westchester Medical Center Health Network, Valhalla, USA; 7 Department of Neurology and Neurosurgery, New York Medical College, New York, USA

**Keywords:** Aneurysm, Subarachnoid hemorrhage, Cerebral vasospasm, Delayed cerebral ischemia, Clazosentan

## Abstract

**Background:**

For patients presenting with an aneurysmal subarachnoid hemorrhage (aSAH), delayed cerebral ischemia (DCI) is a significant cause of morbidity and mortality. The REACT study is designed to assess the safety and efficacy of clazosentan in preventing clinical deterioration due to DCI in patients with aSAH.

**Methods:**

REACT is a prospective, multicenter, randomized phase 3 study that is planned to enroll 400 patients with documented aSAH from a ruptured cerebral aneurysm, randomized 1:1 to 15 mg/hour intravenous clazosentan vs. placebo, in approximately 100 sites and 15 countries. Eligible patients are required to present at hospital admission with CT evidence of significant subarachnoid blood, defined as a thick and diffuse clot that is more than 4 mm in thickness and involves 3 or more basal cisterns. The primary efficacy endpoint is the occurrence of clinical deterioration due to DCI up to 14 days post-study drug initiation. The main secondary endpoint is the occurrence of clinically relevant cerebral infarction at Day 16 post-study drug initiation. Other secondary endpoints include the modified Rankin Scale (mRS) and the Glasgow Outcome Scale-Extended (GOSE) score at Week 12 post-aSAH, dichotomized into poor and good outcome. Radiological results and clinical endpoints are centrally evaluated by independent committees, blinded to treatment allocation. Exploratory efficacy endpoints comprise the assessment of cognition status at 12 weeks and quality of life at 12 and 24 weeks post aSAH.

**Discussion:**

In the REACT study, clazosentan is evaluated on top of standard of care to determine if it reduces the risk of clinical deterioration due to DCI after aSAH. The selection of patients with thick and diffuse clots is intended to assess the benefit/risk profile of clazosentan in a population at high risk of vasospasm-related ischemic complications post-aSAH.

**Trial registration (Additional file 1):**

ClinicalTrials.gov (NCT03585270). EU Clinical Trial Register (EudraCT Number: 2018–000241-39).

**Supplementary Information:**

The online version contains supplementary material available at 10.1186/s12883-022-03002-8.

## Background

Aneurysmal subarachnoid hemorrhage (aSAH) occurs at a rate of 7.9 cases per 100,000 person-years [1] and it is associated with significant morbidity and mortality [2–4]. Despite early aneurysm repair by surgical clipping or endovascular coiling, patients remain at risk of developing cerebral vasospasm characterized by a reduction in the diameter of cerebral arteries [5]. Up to 70% of patients with aSAH develop angiographic vasospasm [6–9]. Clinical deterioration due to delayed cerebral ischemia, so called delayed cerebral ischemia (DCI), has been reported in about 20–50% of patients in early studies [7, 10, 11] and a recent systematic review of aSAH clinical trials has reported a prevalence of 30% with no change observed over time within the last 2 decades [12]. Cerebral vasospasm is a key contributing factor to the progression towards DCI and infarction [8, 13, 14], which leave many survivors with neurologic deficits, reduced quality of life, and cognitive impairment [7, 15].

Hemodynamic therapy consisting of the administration of IV fluids and vasopressors is the first step in the treatment of symptomatic vasospasm [1]. The only approved drug for the prevention of DCI is the calcium channel blocker nimodipine, which has become standard of care in the US and the EU [1, 16]. Nimodipine has been shown to improve neurological outcomes but has little effect on vasospasm [17–19]. For patients with unresponsive severe vasospasm, the main treatment option currently available is endovascular therapy, an invasive procedure with variable clinical success rates [20], consisting of balloon angioplasty for accessible lesions of the proximal larger arteries including the internal carotid, middle cerebral, and basilar arteries, and intra-arterial administration of vasodilators for more distal vessels [1].

The pathogenesis of vasospasm after aSAH is thought to be initiated by clot hemolysis and the release of hemoglobin, oxyhemoglobin, and thrombin in the subarachnoid space resulting in an imbalance between vasoconstrictor (endothelin-1) and vasodilator substances (nitric oxide) [9, 21–23]. Endothelin-1 is one of the most potent vasoconstrictors known [24] and its binding to vasoconstricting receptor subtype A (ETA) has been implicated in the development of cerebral vasospasm following aSAH [25, 26]. Blocking this receptor is a promising therapeutic option that targets the root cause of vasospasm. Since its first description in 1997 [27], clazosentan, a selective ETA receptor antagonist, has been investigated for its anti-vasospastic properties in approximately 2000 patients with aSAH. Four Phase 2, proof of concept studies have investigated the potential of clazosentan to prevent and possibly reverse vasospasm, as well as decrease vasospasm severity [28–31]. A phase 2 dose finding study performed in Japanese and Korean patients and 2 phase 3 global studies have further supported the efficacy of the 10 mg/hour dose in Japanese patients and the 15 mg/hour dose in the global patient population [30, 32, 33]. Two phase 3 studies performed in Japanese patients with aSAH secured by endovascular coiling in one study and surgical clipping in the other [34] subsequently led to the approval of the 10 mg/hour clazosentan dose in Japan, in 2022, for the prevention of cerebral vasospasm, vasospasm-related cerebral infarction, and cerebral ischemic symptoms after aSAH surgery. In parallel, the 15 mg/hour dose was selected based on the results from the global phase 2 and 3 studies for further investigation in the ongoing REACT (pRevention and trEatment of vAsospasm with ClazosenTan) study in patients with a clipped or coiled aneurysm after aSAH. The design of this study is strongly supported by the knowledge that was gained over the years during the clinical development program of clazosentan. Across all clazosentan studies, the treatment effect of clazosentan 10 or 15 mg/hour on the composite vasospasm-related morbidity and all-cause mortality endpoint was driven by the vasospasm-related delayed ischemic neurological deficit component (also called clinical deterioration due to delayed cerebral ischemia). This endpoint which captures the immediate clinical manifestations of post-aSAH cerebral ischemia was selected as the primary endpoint of the REACT study. The previous studies also identified a population of patients who have a high risk for vasospasm-related ischemic complications because of extensive cisternal clot burdens at admission [31, 35]. This finding was used to implement a high-risk patient enrichment strategy in the REACT study to maximize the overall benefit/risk profile. The safety profile of clazosentan has been well-documented in past studies and the adverse events which are commonly reported for selective endothelin-A receptor antagonists (e.g., fluid retention and hypotension) are identified and can be managed following specific treatment guidelines.

The REACT study protocol is detailed in anticipation of the upcoming study results.

## Methods

### Study aim, design, and setting

The primary goal of the REACT study is to determine the efficacy of clazosentan in preventing clinical deterioration due to DCI up to 14 days post-study drug initiation. Secondary objectives include the evaluation of the effect of clazosentan on the occurrence of clinically relevant cerebral infarction at Day 16 post-study drug initiation and the impact on the long-term clinical outcome and cognition at Week 12, and on quality of life at Week 12 and 24 post-aSAH. The assessment of safety and tolerability up to 24 hours after study drug discontinuation is also a secondary objective. In addition, the study evaluates the effect of clazosentan on healthcare resource utilization.

**Fig. 1:**
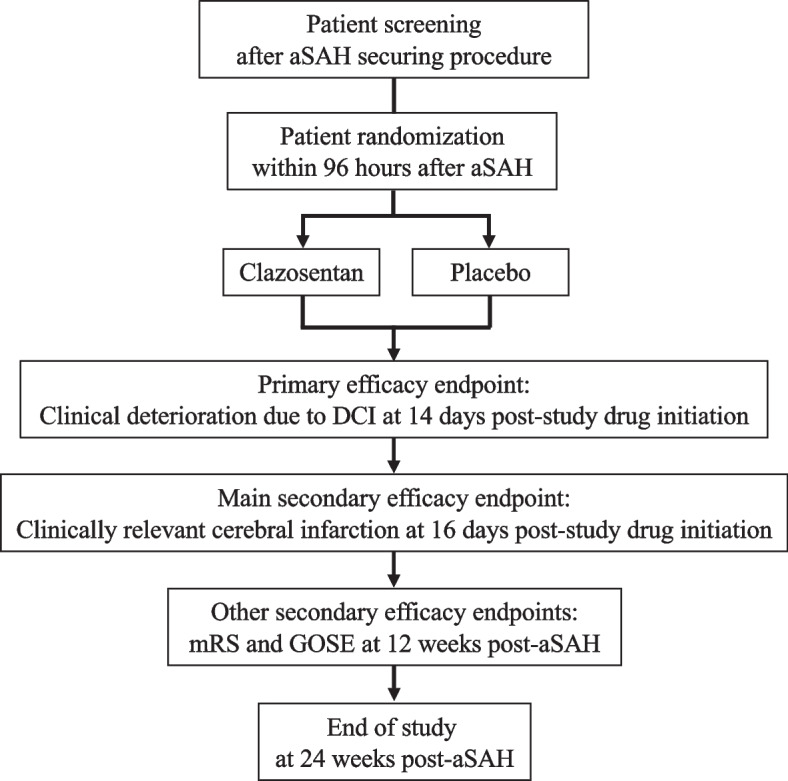
Flow of participants

REACT is a prospective, multicenter, double-blind, randomized, placebo-controlled, parallel-group, phase 3 study. It aims to enroll 400 patients with aSAH from a ruptured brain aneurysm at approximately 100 sites (list available on Clinicaltrials.gov) in approximately 15 countries. After randomization to clazosentan vs. placebo, patients enter a double-blind treatment period for a maximum duration of 14 days, which is followed by a safety follow-up period of 24 hours, preceding any hospital discharge. The extended follow-up period includes an on-site visit 12 weeks after the aSAH, and a telephone interview 24 weeks after the aSAH (end-of-study visit) for safety and efficacy assessments (Fig. 1).

### Study population

The REACT study enrolls a population of patients at high risk of developing cerebral vasospasm and DCI because they are required to have large amounts of subarachnoid blood at hospital admission based upon CT findings. The patients should have thick and diffuse clot, defined as a thick confluent clot, more than 4 mm in thickness, involving 3 or more basal cisterns (Additional file 2) [36]. This enriched population is likely to present with greater vasospasm-related morbidity than the broader aSAH population, which has been investigated so far. A second group of high-risk patients, which included those patients who had already developed vasospasm with no significant neurological deterioration, was initially planned by protocol. However, recruitment into this so-called ‘Early Treatment group’ was discontinued because inclusion rates were low, making the contribution of these patients to the overall study futile (Additional file 3).

The inclusion and exclusion criteria are listed in Table 1 and Table 2, respectively.

**Table 1:** Inclusion criteria of the REACT trial

- Written informed consent to participate in the study must be obtained from the patient or proxy / legal representative at any time from hospital admission to prior to initiation of any study-mandated procedure.	
- Males and females 18 to 70 years of age (inclusive, at hospital admission)	
- Patients with a ruptured saccular aneurysm, angiographically confirmed by DSA or CTA, which has been successfully secured within 72 hours of rupture by surgical clipping or endovascular coiling.	
- WFNS grades 1–4 (based on GCS) assessed after recovery from the aneurysm-securing procedure and after external ventricular drainage for hydrocephalus, if required	
- Presence of a thick and diffuse clot^a^ on the hospital admission CT scan, absence of cerebral vasospasm at the time of randomization, and possibility to start study drug in the ICU (or equivalent environment where all protocol assessments can be performed and the Patient Management Guidelines followed) within 96 hours following the time of the aneurysm rupture.	
- Presence of a cerebral CT scan performed at least 8 hours post aneurysm-securing procedure and within 24 hours prior to randomization. - Absence of a significant (e.g., symptomatic or large) new or worsened cerebral infarct or re-bleeding of the repaired aneurysm on the post-procedure CT scan.	
- A woman of childbearing potential is eligible only if the pregnancy test performed during the screening period is negative. Agreement must be obtained to take the necessary precautions to avoid pregnancy from hospital discharge until 30 days post-study drug discontinuation. If breastfeeding, agreement must be obtained to refrain for the duration of the treatment with study drug and until 30 days post-study drug discontinuation.	
- Males are eligible for study participation only if they agree to take the necessary precautions to avoid pregnancy in a female partner from hospital discharge until 30 days post-study drug discontinuation.	

*CTA* computed tomography (angiogram), *DSA* digital subtraction angiogram, *GCS* Glasgow Coma Scale, *ICU* intensive care unit, *WFNS* World Federation of Neurological Societies

^a^Defined as a thick confluent clot, more than 4 mm in thickness, involving 3 or more basal cisterns

**Table 2:** Exclusion criteria of the REACT trial

aSAH, aneurysm-securing procedure, vasospasm	
- Patients with SAH due to causes other than a saccular aneurysm (e.g., trauma or rupture of fusiform or mycotic aneurysms, SAH associated with arterio-venous malformation, vertebral dissections).	
- Patients with at least one unruptured aneurysm for whom a subsequent intervention is planned within 3 months of the aSAH.	
- Significant bleeding post aneurysm-securing procedure (e.g., due to intra-ventricular drain, intra-cerebral hemorrhage, epidural hematoma, vessel dissection or rupture, re-bleeding of the repaired aneurysm), based on investigator judgment.	
- Intra- or perianeurysm securing procedure complication, requiring non-routine medical or interventional treatment such as administration of an antithrombotic or anti-platelet agent (e.g., abciximab), which is not completely resolved prior to randomization.	
- Intraventricular hemorrhage on the admission CT scan, filling more than 50% of both lateral ventricles and with involvement of the 3rd and 4th ventricles.	
- Intracerebral hemorrhage on the admission CT scan with an approximate volume of > 50 mL.	
- Presence of cerebral vasospasm at hospital admission (initial admission or transfer from another hospital) believed to be associated with a prior bleed (i.e., occurring before the bleed for which the patient is currently hospitalized). Vasospasm occurring during the aneurysm-securing procedure is not an exclusion criterion.	
Neurological and functional status	
- Patients with a new major neurological deficit occurring post aneurysm-securing procedure, which is attributable to the procedure and does not improve to pre-procedure status before randomization.	
- Patients who are still under the influence of pharmacological sedation at the time of randomization or who are, for whatever reason, not evaluable for baseline and regular daily neurological assessments.	
WFNS grade 5 (based on GCS) immediately prior to planned randomization, assessed after external ventricular drainage for hydrocephalus, if required.	
- GCS score ≤ 9 at the time of randomization and without ICP monitoring	
- mRS score ≥ 3 before the aSAH (i.e., due to a chronic condition).	
Other clinical considerations	
- Patients with total bilirubin > 2 × the upper limit of normal, and/or a known diagnosis or clinical suspicion of liver cirrhosis or moderate to severe hepatic impairment.	
- Any concomitant condition or disease (including psychiatric and neurological conditions, drug abuse, severe alcoholism), which, in the opinion of the investigator, would affect the assessment of the safety or efficacy of the study treatment.	
- Hypotension (systolic blood pressure ≤ 90 mmHg) at the time of randomization that is refractory to treatment.	
- Unresolved pulmonary edema or significant pneumonia still present at the time of randomization, or severe hypoxia at the time of randomization in intubated patients defined as PaO_2_/FiO_2_ ≤ 200.	
- High sustained ICP (> 25 mmHg lasting > 20 minutes) at the time of randomization, despite optimal treatment in patients with ICP monitoring.	
- Severe cardiac failure requiring inotropic support at the time of randomization.	
Medications and therapies	
- Known hypersensitivity to clazosentan or any excipient in the formulation.	
At any time from hospital admission to randomization:	
- Lumbar and/or cisternal drainage performed specifically to prevent or treat cerebral vasospasm.	
- Cerebral angioplasty or intra-arterial vasodilators.	
- Intrathecal, intracisternal, or intraventricular thrombolytics.	
- Intra-aortic balloon counter-pulsation devices.	
- Investigational drugs, procedures or devices.	
- Strong inhibitors of OATP1B1 and OATP1B3 transporter proteins (e.g., cyclosporin A, rifampicin, lopinavir/ritonavir).	
Within 4 hours prior to randomization:	
- Intrathecal, intracisternal, and intraventricular vasodilators (e.g., nimodipine), i.v. nicardipine (except for blood pressure control), or i.v. milrinone.	
Anticipated at randomization:	
- Urgent rescue therapy (i.e., cerebral angioplasty, intra-arterial/intrathecal/intracisternal/ intraventricular vasodilators).	
- Strong inhibitors of OATP1B1 and OATP1B3 transporter proteins.	

*aSAH* aneurysmal subarachnoid hemorrhage, *CT* computed tomography, *GCS* Glasgow Coma Scale, *ICP* intracranial pressure, *i.v*. intravenous, *mRS* modified Rankin Scale, *OATP* organic anion transporting polypeptide, *PaO2/FiO2* ratio of arterial oxygen partial pressure to fractional inspired oxygen, *WFNS* World Federation of Neurological Societies

### Patient screening and randomization

The screening period starts with the signature of the informed consent form (Additional file 4) by the patient and/or his/her legally designated representative/proxy. If neither the patient nor his/her legally designated representative/proxy can sign the consent form, a deferral of consent process may be followed if allowed by local regulations and if study-specific approval is previously received. If the patient is not able to provide personal consent at the time the consent is obtained, then he/she must provide this consent as soon as possible once his/her clinical condition has improved to the extent that providing personal consent is possible, unless local regulations state otherwise. The screening period ends with randomization occurring within 96 hours after aSAH. Patients are randomized 1:1 to clazosentan or placebo, and they are stratified by World Federation of Neurological Societies (WFNS) grade (1–2 versus 3–5) and age at hospital admission (≤ 60 and > 60 years), which are both recognized factors for unfavorable outcome [37]. Randomization is performed by the Interactive Response Technology system, which allocates a medication to the patient.

### Study treatments

Patients receive clazosentan or placebo as a continuous 15 mg/hour intravenous (i.v.) infusion for up to 14 days with a minimum of 10 days, on top of the usual standard of care for the management of aSAH. The study drug infusion is administered in an intensive care unit in parallel to the administration of hemodynamic therapy as per the Patient Management Guidelines (Additional file 5). This study is performed in a double-blind fashion; the investigators and the personnel involved in the conduct of the study remain blinded to the study treatment received by the patients during the double-blind treatment period until study closure. The identity of the study treatment may be revealed (through the Interactive Response Technology system) only if the patient experiences an emergency medical event, the management of which would require knowledge of the blinded treatment assignment. Although the decision to unblind resides solely with the investigator, discussion with the Sponsor prior to any unblinding is recommended.

### Study drug interruption/discontinuation

Study drug may be temporarily interrupted in response to an adverse event, a diagnostic or therapeutic procedure, a laboratory abnormality, or for administrative reasons. The Patient Management Guidelines (Additional file 5) also recommend temporarily interrupting study drug when the target blood pressure cannot be achieved (after the discontinuation of nimodipine, if applicable) or when pulmonary ventilation/perfusion ratio mismatch is suspected (or is persistent despite discontinuation of nimodipine, if applicable). Interruptions of study treatment should be kept as short as possible.

Study treatment may be discontinued in response to an adverse event, lack of efficacy (including disease progression, worsening of patient’s condition), a protocol deviation (including eligibility failure, non-compliance with study requirements), a diagnostic or therapeutic procedure, a laboratory abnormality, or for administrative reasons.

### Allowed concomitant therapy

The usual standard of care for the management of aSAH, including oral or i.v. nimodipine, is allowed except for those therapies considered forbidden as described below. “Statins” (e.g., simvastatin, pravastatin) may only be administered if the patient was receiving them chronically for treatment of high cholesterol. Urgent endovascular rescue therapy for the treatment of refractory vasospasm (i.e., balloon angioplasty, intra-arterial/intrathecal/intracisternal/intraventricular vasodilators) can be administered at any time during the treatment period for refractory vasospasm but requires temporary interruption of study drug. Intravenous administration of vasodilators (e.g., nicardipine, milrinone) are allowed as rescue therapy only if preceded by intra-arterial administration of a vasodilator.

Vaccines (including those for Coronavirus disease 2019 [COVID-19]) may be administered at any time during the study.

### Forbidden medications

The following treatments are not allowed until the primary endpoint is assessed due to their potential to interfere with the evaluation of efficacy or safety, or due to the potential for a drug-drug interaction with study drug: intra-aortic balloon device; lumbar and/or cisternal drainage for the prevention of cerebral vasospasm and/or DCI; i.v. milrinone, i.v. nicardipine (may be used for blood pressure control), and intrathecal/intra-cisternal/intra-ventricular vasodilators (e.g., nimodipine); i.v. magnesium, i.v. albumin, or plasma volume expander if administered specifically for the prevention of vasospasm and/or DCI; thrombolytics (except to open an occluded drain) and antifibrinolytics; i.v. hypertonic saline (in the absence of hyponatremia, brain edema, or high intracranial pressure [ICP]); i.v. mannitol (in the absence of brain edema or high ICP); strong inhibitors of organic anion transporting polypeptide (OATP)1B1 and OATP1B3 transporter proteins; other endothelin receptor antagonists; any investigational drugs, procedures, or devices; traditional medicines.

### Study endpoints

#### Primary endpoint

The primary efficacy endpoint (adjudicated by an independent Clinical Event Committee (CEC)) is the occurrence of clinical deterioration due to DCI, from study drug initiation up to 14 days post-study drug initiation. Clinical deterioration due to DCI is defined as a worsening of at least 2 points on the modified Glasgow Coma Scale (mGCS) or the abbreviated National Institutes of Health Stroke Scale (aNIHSS), lasting for at least 2 hours, which cannot be entirely attributed to causes other than cerebral vasospasm (Appendices 3 and 4).

Patients who die between study drug start up to and including 14 days post-study drug initiation are considered as presenting with clinical deterioration due to DCI. Patients who cannot be evaluated for neurological status at any time during this same period are considered as presenting with clinical deterioration due to DCI if either rescue therapy was administered for a relevant vasospasm or the reason for not being evaluable is vasospasm related.

Patients who are discharged from the study site prior to Day 14 post-study drug initiation have a follow-up visit or telephone call, covering their clinical status between discharge and Day 14 post-study drug start. If this follow-up reveals that the patient was re-hospitalized or transferred to another facility, and DCI cannot be ruled out as a primary or contributing cause, the patient is considered as meeting the primary endpoint. If a patient has less than 14 days of neurological scales available and there is an absence of follow-up information (including patients withdrawn from the study during the observation period) the primary endpoint is assessed based on the totality of available clinical data.

#### Secondary endpoints

The main secondary endpoint is the occurrence of clinically relevant cerebral infarction at Day 16 post-study drug initiation defined as all cause cerebral infarction ≥ 5 cm^3^ or cerebral infarction < 5 cm^3^ in patients with clinical deterioration due to DCI. This definition was updated following protocol amendment (Additional file 3).

Cerebral infarction refers to new or worsened infarcts and is determined by central radiology review, comparing the total volume of infarcts on the CT scan performed 16 days after study drug initiation with the total volume on the CT scan performed just prior to randomization. If the CT scan cannot be performed on Day 16, then it is acceptable for the CT to be performed within 7 days following Day 16. If a patient is discharged from the hospital prior to Day 16, the CT scan is performed on the day of hospital discharge. Clinical deterioration due to DCI and cerebral infarctions ≥ 5 cm^3^ are confirmed by the CEC. Cerebral infarctions < 5 cm^3^ in patients with clinical deterioration due to DCI are derived from both the CEC data (primary endpoint) and the Independent Radiology Committee (IRC) data (for infarct size).

Other secondary endpoints include long-term clinical outcome, assessed at Week 12 post-aSAH by the modified Rankin scale (mRS) [38] dichotomized into poor outcome (score ≥ 3) and good outcome (score < 3) and the Glasgow Outcome Scale-Extended (GOSE) [39], dichotomized into poor outcome (score ≤ 4) and good outcome (score > 4).

#### Exploratory efficacy endpoints

Exploratory endpoints include cognitive status assessed with the Montreal Cognitive Assessment (MoCA; values at Day 14 after study drug initiation and at Week 12 post-aSAH, changes from baseline to Day 14 and to Week 12, and changes from Day 14 to Week 12).

Other efficacy endpoints include the number of episodes of clinical deterioration due to DCI from study drug initiation up to 14 days post-study drug initiation, and the occurrence of all-cause new or worsened cerebral infarction ≥ 5 cm^3^ in total volume (initially planned as main secondary endpoint, see Additional file 3), as adjudicated by the CEC.

#### Quality of life endpoints

Quality of life endpoints are assessed with 2 generic instruments including the EQ-5D (values at Week 12 post-aSAH, at Week 24, and change in index score and visual analog scale from Week 12 to Week 24) and the Oxford Participation and Activities Questionnaire (Ox-PAQ; values at Week 12). Quality of life is also evaluated with the Stroke Specific Quality of Life (SS-QOL; values at Week 12).

#### Pharmaco-economic endpoints

The pharmaco-economic endpoints describe the number and type of episodes of rescue therapy, and the number and type of specific medical treatments and therapies from randomization up to hospital discharge and from hospital discharge up to Week 12 post-aSAH. The length of initial and total intensive care unit stay, the length of total hospitalization, and duration in different hospital/institutional care units are recorded from randomization up to hospital discharge and from hospital discharge up to Week 12. The intensity of rehabilitation care up to Week 12 post-aSAH, the first post-hospital discharge location, and the duration of home care support post-initial hospital discharge are documented. The employment status is collected at Week 24 post-aSAH.

#### Biomarker endpoints

The area under the plasma concentration-time curve of the S100b protein from baseline to Day 10 and 14 post-study drug initiation is to be calculated. The S100b protein has emerged as a brain ischemia biomarker that is implicated in the pathogenic process of aSAH [40].

#### Safety endpoints

The safety endpoints include the occurrence of treatment-emergent adverse events (TEAEs), serious TEAEs, TEAEs of specific interest (i.e. pulmonary complications, hypotension, anemia, cerebral hemorrhage, cerebral edema, fluid retention, hepatic disorders, tachyarrhythmia), and treatment-emergent marked laboratory abnormalities up to 24 hours after study drug discontinuation (along with changes from baseline to end of study drug administration for selected centrally assessed laboratory parameters). TEAEs leading to premature discontinuation of study drug and death (all causes) up to week 24 post-aSAH are to be collected. The occurrence of rescue therapy-specific adverse events up to hospital discharge (or up to week 12, whichever is earlier) is also documented.

### Study assessments

An overview of the timing of the assessments is presented in Table 3. For those patients who cannot return to the investigational site for the 12-week visit, the GOSE and quality of life assessments are conducted by telephone and post, respectively. Depending on local regulations, a study staff member from the investigative site may also conduct the week 12 visit at the patient’s place of residence. If the 24-week (end-of-study) visit cannot be conducted as a telephone interview, the patient is asked to return the completed data collection forms by post.

**Table 3:** Overview of study assessments

PERIOD	Hospital admission	SCREENING Period		OBSERVATION Period (for 14 days post-study drug initiation irrespective of treatment duration)				24 h safety FU Period	Extended FU Period (From end of 24 h safety FU Period until EOS)	
			TREATMENT Period (min. 10, max. 14 days of treatment)							
Timing / assessment		within 96 hours post-aSAH			for 14 days post-SD start			until 24 h post-SD stop	WEEK 12 VISIT	END OF STUDY (EOS)^s^
		From ICF to randomization	Prior to study drug (SD) start	SD start	Daily in ICU^h^	During observation period^h^	Worsening of ≥2 points on mGCS / aNIHSS^h^	End-of-Treatment (EOT)	84 days post-aSAH (± 7 d)	24 weeks (168 days ±14 days) post-aSAH
Informed consent		**X**								
Demographics		**X**								
Medical history		**X**	**X**							
Incl./Excl. criteria		**X**								
Height, weight		**X**								
Vital signs (BP, HR, ICP^a^, CVP^a^)			**X (within 60 min)**		**q6h (± 1 h)**	**q6h (± 1 h) (every 12 h if not in ICU)**				
Body temperature			**X (within 60 min)**		**X (every 12 h ± 1 h)**					
Fluid balance (24 h)^p^			**X**		**X**					
ECG parameters			**X (within 60 min)**		**X**^i^			**X (within 2 h post-SD stop)**		
Laboratory tests (local [l]/ central [c])		**X (l)**	**X (c) (within 60 min)**		**X**^j^ **(c) (EOD for 14 days)**		**X (l) / SpO**_2_	**X**^j^ **(c) (within 2 h post-SD stop)**		
Biomarker			**X (c) (within 60 min)**		**X (c) (EOD for 14 days)**		**X (c) if CNS cause**	**X (c) (within 2 h post-SD stop)**		
Pregnancy test		**X (serum, (l))**							**X (urine)**	
Concomitant medications^r^	**X**				**X**					
Non-drug treatments / interventions			**X**		**X**					
WFNS	**X**^b^	**X, X**^c^								
Total GCS	**X**^b^	**X, X**^c^								
mGCS/aNIHSS			**X (within 30 min)**		**q6h**^k^ **(± 1 h)**	**X**^l^ **(± 1 h)**	**X (hourly ± 15 min for first 2 h)**			
Angiogram (DSA or CTA)	**X**^d^	**X (local standard of care, not assessed centrally)**					**X (if CNS cause)**			
CT scan	**X**^d^	**X**^e^					**X (if CNS cause)**	**X**^m^ **(16 days post-SD start)**		
Subject narrative							**X (14 days post-SD start)**			
MoCA^f^		**X**^g^				**X (14 ± 1 day post-SD start)**^q^			**X**	
GOSE									**X**	
SS-QOL, Ox-PAQ									**X**	
EQ-5D									**X**	**X**
Study drug administration				**X**	**X**					
Adverse events^n^		**X**			**X**					
Serious adverse events^o^		**X**			**X**					
Pharmaco-economic assessments			**X**		**X**					
Employment status										**X**

*AE* adverse event, *aNIHSS* abbreviated National Institutes of Health Stroke Scale, *aSAH* aneurysmal subarachnoid hemorrhage, *BP* blood pressure, *CNS* central nervous system, *CT* computerized tomography, *CTA* computerized tomography angiography, *CVP* central venous pressure, *DSA* digital subtraction angiography, *ECG* electrocardiogram, *eCRF* electronic case report form, *EOD* every other day, *EOS* End-of-Study, *EOT* End-of-Treatment, *FU* follow-up, *GCS* Glasgow Coma Scale, *GOSE* Glasgow Outcome Scale Extended, *HR* heart rate, *ICF* informed consent form, *ICP* intracranial pressure, *ICU* intensive care unit, *mGCS* modified Glasgow Coma Scale, *MoCA* Montreal Cognitive Assessment, *Ox-PAQ* Oxford Participation and Activities Questionnaire, *SAE* serious adverse event, *SD* study drug, *SpO*﻿_2_ peripheral capillary oxygen saturation, *SS-QOL* Stroke Specific Quality of Life, *WFNS* World Federation of Neurological Societies

^a^ICP/CVP is measured and recorded for those patients with ICP and/or CVP monitoring in place

^b^If the patient was transferred from another hospital, the GCS score and WFNS grade correspond to the assessments made at the referral hospital, unless these were not done or not reliable

^c^Two assessments: post aneurysm-securing procedure and prior to randomization

^d^If performed at a referral hospital, is of acceptable quality, and is available in digital format at the investigational site at the time of screening, does not need to be repeated

^e^This CT scan is to be performed at least 8 hours after the aneurysm-securing procedure and within 24 hours prior to randomization

^f^Only performed if patient is GCS ≥ 13 and extubated (if applicable)

^g^As soon as possible after recovering from the aneurysm-securing procedure and prior to SD start

^h^If there is a worsening of at least 2 points in the mGCS and/or the aNIHSS the assessments in the “worsening” column must be performed on top of the regularly scheduled assessments. The mGCS and the aNIHSS must be repeated hourly for at least the first 2 hours after a 2-point worsening. If the deterioration is believed to be of CNS origin, a cerebral angiogram and a cerebral CT scan must be performed within 6 hours of the start of the symptoms and submitted for central review and a blood sample for S100b protein must be drawn within 1 hour of the confirmation of the neurological deterioration episode or no later than 3 hours from the initial worsening. Local lab tests should be obtained as close as possible to the time of the clinical worsening (max. 1 hour after time of confirmed worsening)

^i^QT, QRS, PR, RR intervals, and HR are measured and recorded in the eCRF if patient experiences an AE related to cardiac rhythm abnormalities

^j^Any clinically significant laboratory values must be reported as an AE/SAE as appropriate and those still abnormal at the time of the EOS assessment are followed up based on local routine standard of care. A local laboratory may be requested by the sponsor to document the event and its resolution, and the results recorded in the eCRF

^k^At least once per day for patients that require uninterrupted continuous sedation

^l^After the end of the study drug infusion, the mGCS and aNIHSS continue to be assessed every 6 hours if the patient is still in the ICU (or equivalent ward), until 14 days after study drug initiation. They are assessed at least once per day if the patient requires continuous uninterrupted sedation. If the patient is no longer in the ICU (i.e., has been sent to a regular/general ward), the mGCS and aNIHSS are assessed at least once every 12 hours (± 1 h) until 14 days after study drug initiation. In the unavoidable situation where the patient is discharged from the study site before completing the observation period, their clinical status must be followed-up to cover the period between discharge and Day 14 post-study drug start. The follow-up should be performed on Day 14 post-study drug initiation or as soon as possible after. This follow-up is not required if the patient was discharged on Day 13 and there is at least one set of neurological assessment scales available on this day

^m^If the CT scan cannot be performed on the 16th day post-SD start, then it is acceptable if the CT scan is performed up to 7 days after Day 16. The CT scan is performed on the day of hospital discharge for those patients who are discharged from the hospital prior to 16 days after study drug start. If no CT scan is available at hospital discharge, the last CT scan performed prior to discharge may be used for this assessment

^n^All AEs that occur after signing the ICF and up to the EOS visit must be recorded if related to a study-mandated procedure. All other AEs are to be reported from SD initiation until 24 hours post-permanent SD discontinuation

^o^All SAEs that occur after signing the ICF and up to the EOS visit must be recorded if related to a study-mandated procedure. All other SAEs are to be reported from SD initiation until EOS. Waived SAEs do not require reporting to the sponsor’s Drug Safety department within 24 hours of the knowledge of its occurrence

^p^Applicable during study drug administration only. Balance is captured if a urine catheter is present. Otherwise, 24-hour fluid intake is measured and recorded

^q^This MoCA is performed on the day of hospital discharge for those patients who are discharged from the hospital prior to 14 days after study drug start

^r^For details on the concomitant medication recording refer to text

^s^The EOS visit is conducted remotely as a telephone interview

#### Glasgow Coma Scale (GCS) and modified Glasgow Coma Scale (mGCS)

The GCS (Additional file 6) describes levels of consciousness by testing eye opening, verbal, and motor response [41]. The highest score (fully awake) is 15 and the lowest 3. If the motor response in the left and the right arm are not the same, then the best score out of the two scores is used to determine the total GCS score. The mGCS (Additional file 6) is used in conjunction with the aNIHSS (see below) to detect episodes of clinical deterioration due to DCI [42, 43]. It is performed the same way as the GCS. However, the worst motor response is used to enable the detection of any new focal deficits from one assessment to the next.

#### Abbreviated National Institutes of Health Stroke Scale (aNIHSS)

The aNIHSS (Additional file 7) is a measure of limb movement and strength and it only includes the motor section of the full NIHSS, which is a tool used to objectively quantify the impairment caused by a stroke [44]. Four separate scores from 0 (best) to 4 (worst) are determined for each limb. The total score ranges from 0 (best) to 16 (worst).

#### Glasgow Outcome Scale-Extended (GOSE) and modified Rankin Scale (mRS)

The combined GOSE /mRS structured interview (Additional file 8) measures functional outcome and dependency with minimum bias and high inter-rater reliability [38, 39]. The GOSE score ranges from 1 (dead) to 8 (upper good recovery) and the mRS score from 0 (no symptoms) to 6 (dead).

#### World Federation of Neurological Societies (WFNS) grade

The WFNS grade is a clinical measure of disease severity; it is determined from the GCS score and the presence of motor deficit [45].

#### Montreal Cognitive Assessment (MoCA)

The MoCA is a brief screening assessment for detecting cognitive impairment [46], which has been used successfully in the intensive care unit in alert patients with aSAH (GCS ≥ 13) [47]. It assesses the domains of attention and concentration, executive functions, memory, language, visual-constructional skills, conceptual thinking, calculations, and orientation. The scores range from 0 (worst) to 30 (best). MoCA© Version 7.1 is used. If it is impossible for a patient to return to the investigational site for the week 12 visit the MoCA is not performed.

#### Stroke Specific Quality of Life SS-QOL

The SS-QOL is a patient-reported outcome measure developed to provide an assessment of health-related quality of life specific to patients with stroke [48], which has been validated in patients with aSAH [49]. It includes 49 items that cover 12 domains (energy, upper extremity function, work/productivity, mood, self-care, social roles, family roles, vision, language, thinking, and personality) and 13 questions comparing post-aSAH status with pre-aSAH status. The SS-QOL yields both domain scores and an overall summary score. SS-QOL© V2.0 are used. If the patient is unable to complete the questionnaire, a proxy (e.g., family member, caregiver, close friend) is asked to complete the questionnaire.

#### Oxford Participation and Activities Questionnaire (Ox-PAQ)

The Ox-PAQ is a patient-reported outcome measure, which assesses generic health-related quality of life [50]. The instrument evaluates the ability of individuals to engage in activities (such as work, hobbies, daily routines) and the level of dependency an individual has on others. It is comprised of 23 questions that cover 3 domains (routine activities, emotional well-being, and social engagement). If the patient is unable to complete the questionnaire it is not completed by a proxy.

#### EQ-5D

The EQ-5D is a patient-reported outcome measure developed to assess generic health-related quality of life [51]. It is composed of 5 domains (mobility, self-care, usual activities, pain/discomfort, anxiety/depression) and a visual analog scale assessing overall health. The 5L version is used. If the patient is unable to complete the questionnaire, a proxy (e.g., family member, caregiver, close friend) is asked to complete the questionnaire.

#### Safety assessments

Height, weight, vital signs including intracranial pressure and central venous pressure (if monitored), body temperature, fluid balance, electrocardiogram parameters, laboratory test results are collected according to the schedule provided in Table 3. In case of episodes of clinical deterioration, a blood sample is drawn as close as possible to the time of the initial neurological deterioration, but no later than 1 hour after the confirmation of the deterioration or within 3 hours after the initial worsening. Serum sodium, creatinine, and arterial blood gases (arterial oxygen saturation, partial pressure of carbon dioxide and oxygen in arterial blood) and pH (if the patient is intubated/ventilated) must be included as a minimum. Adverse events and serious adverse events are collected throughout the study.

### Statistical analysis

#### Statistical hypotheses

Four null hypotheses are tested according to a fixed sequence procedure (primary endpoint, main secondary endpoint, other secondary endpoints -mRS then GOSE-) at the two-sided significance level of 0.05 until first non-rejection.

#### Analyses of the study variables

The primary statistical analysis is performed on the full analysis set, which includes all patients from the randomized analysis set who have started the study treatment. This is in accordance with the intention-to-treat principle as a) randomized but untreated patients are rare and b) the decision whether or not to begin treatment cannot be influenced by knowledge of the assigned treatment. The primary endpoint is analyzed using a Cochran-Mantel-Haenszel test, stratified on WFNS grade (1–2 versus 3–5) and age at hospital admission (≤ 60 and > 60 years). The treatment effect (clazosentan versus placebo) is expressed in terms of odds ratios and also in terms of relative risk reduction of the active arm compared to placebo with corresponding 95% confidence limits.

Supportive analyses include logistic regression to estimate the treatment effect after adjusting for WFNS grade and age at hospital admission. Subgroup analyses are conducted for WFNS grade and age at hospital admission.

Similar analyses are performed for the primary and secondary efficacy endpoints.

The safety analysis provides descriptive statistics for each treatment arm.

No interim analyses are planned in this study.

#### Sample size

The sample size is derived from the assumption that the true incidence of clinical deterioration due to DCI up to 14 days after study drug initiation is 28% in the placebo arm and 14% in the clazosentan arm [35]; a sample size of 176 patients in each treatment arm has 90% power to show the superiority in response of clazosentan compared to placebo using Pearson’s χ^2^ test with a 5% two-sided significance level. When taking an approximate 10% drop-out rate into account, 400 patients have to be enrolled in the study, with 200 patients randomized to each treatment arm.

#### Handling of missing data

The CEC provides a final assessment (yes or no) on the primary endpoint, indicating for each patient whether it has been met, therefore it is assumed that there are no missing data for the primary endpoint. In addition, the CEC distinguishes between the cases of clinical deterioration due to DCI and the cases imputed according to the substitution rules described for the primary endpoint.

The CEC provides a final assessment (yes or no) on the first component of the main secondary endpoint (infarcts ≥ 5 cm^3^), indicating for each patient whether it has been met and distinguishing between the true cases of cerebral infarction ≥ 5 cm^3^ and imputed cases (as per CEC charter). Regarding the second component, patients who met the primary endpoint as per CEC but with missing CT scan at Day 16 are considered to have met the secondary endpoint (i.e., the worst possible outcome is assumed).

### Changes to the protocol

Changes to the protocol occurring after study start are listed in Additional file 3. Most importantly, the main secondary endpoint definition was updated to include clinically relevant infarcts < 5 cm^3^ in addition to all-cause infarcts ≥ 5 cm^3^ (as initially planned) at day 16 post-study drug initiation. The latter is to be analyzed as an exploratory endpoint. The mRS was formally included in the statistical hierarchical testing strategy, just before the GOSE.

### Exceptional measures to ensure patient safety and counteract potential trial conduct disruption due to the COVID-19 pandemic

As a consequence of the Coronavirus disease 2019 (COVID-19) declared a pandemic by the World Health Organization (WHO) on 11 March 2020, Idorsia put the recruitment for the REACT study on hold temporarily, on 19 March 2020.

All protocol deviations related to the COVID-19 crisis are to be identified and tracked. This will allow, at the end of the trial, a reconstruction of the impact that such deviations had on the trial integrity and interpretability.

In case of logistical restrictions due to the COVID-19 pandemic, it is possible to postpone the Week 12 assessments up to Week 24 (at the latest) and to perform the safety follow-up of ongoing adverse event in an alternative hospital or local laboratory. It is reminded that if OATP inhibitors are used (e.g., for the treatment of a patient with COVID 19) then study drug must be permanently discontinued as per study protocol. It is possible to perform remote monitoring and source data verification if allowed locally, otherwise alternatives may be agreed with the principal investigator to ensure data integrity.

### Study committees

An Independent Data Monitoring Committee is responsible for monitoring unblinded safety and efficacy data at regular intervals.

An Independent Radiology Committee (IRC) composed of radiologists, who are independent from the study sponsor and blinded to treatment allocation, review all angiograms and CT scans to document and quantify specific radiological findings including clot size, cerebral vasospasm, and cerebral infarction.

An Independent Clinical Event Committee (CEC) composed of clinicians with expertise in aSAH, who are blinded to treatment allocation, determine whether the primary endpoint and the main secondary endpoints have been met. This committee reviews clinical and imaging data from all patients to determine which cases fulfill the definition of clinical deterioration due to DCI. It also adjudicates cases for the presence of a new or worsened cerebral infarct ≥ 5 cm^3^ based on the central review of CT scans performed by the IRC or other available data when the CT scan is missing at day 16 post-study drug initiation. Cerebral infarcts < 5 cm^3^ in patients with clinical deterioration due to DCI are derived from both the CEC data (primary endpoint) and IRC data (for infarct size).

Committee charters are available from the corresponding author upon reasonable request once trial results are published.

### Monitoring

Data from source documents are reported in the patient electronic case report form using electronic data capture. Results from laboratory analyses are electronically sent to the sponsor. Adverse events and medical history are coded according to the latest Medical Dictionary for Regulatory Activities (MedDRA™) version used by the sponsor. Medications are coded according to the latest WHO Drug Dictionary version used by the sponsor.

All data and documentation related to the trial are stored on site for as long as is necessary to comply with the sponsor’s requirements and national and/or international regulations. Access to data is restricted to authorized trial personnel.

The sponsor representatives may audit the investigator site.

### Study status

The planned recruitment of 400 patients was reached in May 2022 and the study was completed in November 2022.

## Discussion

Delayed cerebral ischemia contributes significantly to poor outcome after aSAH [15] and there is a high medical need for improved treatment options to avoid brain infarction, neurological complications and the need for additional endovascular therapy. Clazosentan has been shown to reduce the incidence and severity of cerebral vasospasm and to decrease vasospasm-related morbidity at 6 weeks, in patients with aSAH [29, 30, 32–34]. The effect of clazosentan on vasospasm-related morbidity is particularly noticeable in the patients who present with large amounts of blood clots on the initial CT scan [35], which has been associated with an increased risk for DCI and cerebral infarction [36]. The objective of this ongoing, multicenter, randomized REACT study is to focus on in-hospital clinical deterioration due to DCI, assessed by central adjudication, in an enriched high-risk population including patients with thick and diffuse blood clots at admission. The clazosentan development program has shown that the clinical effect of clazosentan on vasospasm-related events is dose dependent and the 15 mg/hour dose, which was identified in global studies [29, 33] as the most efficacious dose when compared with the 1 and 5 mg/hour doses, is to be evaluated in the REACT trial.

The primary efficacy endpoint of the trial is the occurrence of clinical deterioration due to DCI, from study drug initiation up to 14 days post-study drug initiation. It is defined as a sustained worsening in neurological status that cannot be entirely attributed to causes other than cerebral vasospasm consistent with the recommendations of international multi-disciplinary aSAH research groups [42, 43]. The extension of the duration of the deterioration to 2 hours in the REACT protocol excludes transient fluctuations in clinical status and thus increases the robustness of the endpoint definition. The assessment period for the primary endpoint extends until 14 days after the initiation of treatment, thus covering the treatment period, and corresponding to the period during which DCI is most likely to occur. The proposed primary endpoint captures the most important clinical manifestations of post-aSAH cerebral ischemia that can be prevented by an anti-vasospastic treatment. This clinically relevant endpoint is predictive of cognitive impairment, quality of life deterioration, and poor long-term outcome [7, 15, 52–54]. Therefore, showing a significant reduction in the incidence of clinical deterioration due to DCI, supported by clinically relevant effects on the secondary and exploratory endpoints, is expected to demonstrate the clinically meaningful benefit of clazosentan in this disease indication.

The main secondary endpoint in the REACT study is the occurrence of clinically relevant cerebral infarction, a known complication of aSAH, which has been repeatedly shown to be a strong predictor of poor long-term clinical outcome [7, 55, 56]. A post-hoc analysis of the data from the CONSCIOUS-2 and CONSCIOUS-3 studies demonstrated that infarcts with a total cumulative volume ≥ 5 cm^3^ are mostly related to vasospasm and have a high association with poor clinical outcome at 3 months, as compared to those with a cumulative volume less than 5 cm^3^ [57]. Therefore, the secondary endpoint definition as initially defined (all-cause new or worsened cerebral infarction of a total volume ≥ 5 cm^3^) only included those infarcts ≥ 5 cm^3^ in order to set a meaningful cut-off for infarct volume when considering all infarcts irrespective of cause. Since the determination of underlying infarct etiology based on CT scan assessment is often challenging, the 5 cm^3^ threshold serves as a proxy for vasospasm-related stroke. This endpoint also allowed the identification of ischemic events that are not detectable on clinical examination or that develop in patients who cannot be evaluated neurologically (e.g., due to sedation or very poor clinical status). However, routine blinded monitoring of the event rate during the REACT study revealed a lower-than-expected incidence of infarcts ≥ 5 cm^3^, resulting in insufficient power to detect a treatment effect. This led to an expansion of the endpoint definition to include cerebral infarcts < 5 cm^3^ if they occur in patients with clinical deterioration due to DCI. Although the infarcts < 5 cm^3^ have a lower association with vasospasm and prognosis, their vasospastic origin and their contribution to poor outcome cannot be completely excluded [58, 59]. Therefore, they are included in the secondary endpoint as long as DCI is present to avoid the inclusion of irrelevant infarcts, causing dilution of the treatment effect, as seen in the CONSCIOUS-1 trial [29].

Despite being correlated with the primary efficacy endpoint, the main secondary endpoint goes beyond clinical symptoms since deterioration due to DCI does not always result in the development of cerebral infarction. Conversely, cerebral infarction may be observed on a CT scan in the absence of clinical symptoms in up to 20% of aSAH patients [10, 60]. The assessment of this endpoint at day 16 post-study drug initiation allows for the detection of infarcts that may be the consequence of cerebral ischemia that occur up to day 14 (primary endpoint evaluation is up to day 14).

The other secondary endpoints of the REACT study are the mRS and GOSE scores at Week 12, which assess long-term clinical outcome. These scores have been chosen as secondary, rather than primary efficacy endpoints, because they are strongly driven by the initial hemorrhage and the complications of the aneurysm treatment procedure [61], on which clazosentan has no expected impact. However, the absence of a negative trend on these assessments would be an important part of the overall benefit/risk assessment of clazosentan in patients with aSAH. This justifies the use of mRS and GOSE as other secondary endpoints. The mRS has recently been recommended over the GOSE as the preferred scale for measuring the long-term clinical outcome of SAH by the international clinical experts of the SAH common data elements working group [62]. Therefore, in REACT, the mRS was raised above the GOSE in the statistical hierarchical testing strategy, after protocol amendment (Additional file 3).

Finally, the REACT protocol includes a series of cognitive tests and patient reported outcome tools performed at 12 and/or 24 weeks after the aSAH with the objective to capture most of the aSAH-associated long-term disability.

A key feature of the REACT study is the enrolment of a selected population with a high risk of developing vasospasm-related ischemic complications. This risk is correlated with the amount of blood on the initial CT scan [63]. Thus, the inclusion of patients who present with thick and diffuse clot at hospital admission is expected to increase the occurrence of clinical deterioration due to DCI, allowing the conduct of the study with a reasonable sample size. In the post-hoc analyses of the placebo arm of the CONSCIOUS-2, and CONSCIOUS-3 studies, the patients with thick and diffuse clot at admission, who represented approximately 50% of the overall patient population, had significantly increased risks for DCI and cerebral infarction (relative risk: 2.6 and 2.3, respectively, after adjustment for WFNS at admission) compared with the patients who had a lower amount of blood on the initial CT scan [36]. These high-risk patients benefited most from clazosentan treatment and their relative risk for clinical deterioration due to DCI was significantly reduced by 57% compared with placebo [35]. The REACT study design, which is based on an enriched population, focuses on those patients who are most likely to benefit from clazosentan. In addition, exclusion of patients with hypotension or hypoxia and protocol guidelines on hemodynamic management should reduce the frequency of clazosentan-related main adverse effects. Thus, the REACT study aims at assessing the benefit/risk profile of clazosentan in patients at high risk of vasospasm-related ischemic complications post-aSAH.

## Conclusion

Clazosentan is known to reduce the occurrence and severity of vasospasm occurring after aSAH and was recently shown to decrease the combined incidence of vasospasm-related morbidity and all-cause mortality in two phase 3 studies conducted in Japan. The ongoing REACT study further investigates whether clazosentan can prevent the clinical deteriorations due to DCI and improve long-term outcome in an enriched patient population at high risk of vasospasm-related ischemic events.

## Supplementary Information

**Additional file 1. **Trial registration data.

**Additional file 2. **Definition of “thick and diffuse clot” on hospital admission CT scan.

**Additional file 3. **Changes to the protocol occurring after study start.

**Additional file 4. **Participant information and consent forms.

**Additional file 5. **Patient Management Guidelines.

**Additional file 6. **Glasgow Coma Scale and Modified Glasgow Coma Scale.

**Additional file 7. **Abbreviated National Institutes of Health Stroke Scale.

**Additional file 8. **Glasgow Outcome Scale–Extended and Modified Rankin Scale.

## Data Availability

After completion of the study, the sponsor will post the summary of results within the required timelines on publicly accessible databases, as required by law and regulation. The study protocol and statistical analysis plan will be available at ClinicalTrials.gov. The results of the study will be submitted for publication in a peer-reviewed journal. Authors of supported publications involving the REACT trial will have full access to available study data. There are currently no plans to share individual patient data.

## Abbreviations

aNIHSS: Abbreviated National Institutes of Health Stroke Scale
aSAH: Aneurysmal subarachnoid hemorrhage
CONSCIOUS: Clazosentan to Overcome Neurological Ischemia and Infarction Occurring After Subarachnoid Hemorrhage
COVID-19: Coronavirus disease 2019
CTA: Computed tomography
DCI: Delayed cerebral ischemia
GOSE: Glasgow Outcome Scale-Extended
ICP: Intracranial pressure
i.v.: intravenous
(m)GCS: (Modified) Glasgow Coma Scale
mRS: Modified Rankin Scale
MoCA: Montreal Cognitive Assessment
OATP: Organic anion transporting polypeptide
Ox-PAQ: Oxford Participation and Activities Questionnaire
REACT: PRevention and trEatment of vAsospasm with ClazosenTan
SS-QOL: Stroke Specific Quality of Life
TEAEs: Treatment-emergent adverse events
WFNS: World Federation of Neurological Societies
WHO: World Health Organization

## References

[1] Connolly ES, Rabinstein AA, Carhuapoma JR, Derdeyn CP, Dion J, Higashida RT (2012). Guidelines for the management of aneurysmal subarachnoid hemorrhage: a guideline for healthcare professionals from the American Heart Association/American Stroke Association. Stroke.

[2] Nieuwkamp DJ, Setz LE, Algra A, Linn FH, de Rooij NK, Rinkel GJ (2009). Changes in case fatality of aneurysmal subarachnoid haemorrhage over time, according to age, sex, and region: a meta-analysis. Lancet Neurol.

[3] Lovelock CE, Rinkel GJ, Rothwell PM (2010). Time trends in outcome of subarachnoid hemorrhage: population-based study and systematic review. Neurology.

[4] Al-Khindi T, Macdonald RL, Schweizer TA (2010). Cognitive and functional outcome after aneurysmal subarachnoid hemorrhage. Stroke.

[5] Weir B, Grace M, Hansen J, Rothberg C (1978). Time course of vasospasm in man. J Neurosurg.

[6] Dorsch NW, King MT (1994). A review of cerebral vasospasm in aneurysmal subarachnoid haemorrhage part I: incidence and effects. J Clin Neurosci.

[7] Frontera JA, Fernandez A, Schmidt JM, Claassen J, Wartenberg KE, Badjatia N (2009). Defining vasospasm after subarachnoid hemorrhage: what is the most clinically relevant definition?. Stroke.

[8] Crowley RW, Medel R, Dumont AS, Ilodigwe D, Kassell NF, Mayer SA (2011). Angiographic vasospasm is strongly correlated with cerebral infarction after subarachnoid hemorrhage. Stroke.

[9] Macdonald RL (2014). Delayed neurological deterioration after subarachnoid haemorrhage. Nat Rev Neurol.

[10] Rabinstein AA, Weigand S, Atkinson JL, Wijdicks EF (2005). Patterns of cerebral infarction in aneurysmal subarachnoid hemorrhage. Stroke.

[11] Dankbaar JW, Rijsdijk M, van der Schaaf IC, Velthuis BK, Wermer MJ, Rinkel GJ (2009). Relationship between vasospasm, cerebral perfusion, and delayed cerebral ischemia after aneurysmal subarachnoid hemorrhage. Neuroradiology.

[12] Rigante L, van Lieshout JH, Vergouwen MDI, van Griensven CHS, Vart P, van der Loo L (2022). Time trends in the risk of delayed cerebral ischemia after subarachnoid hemorrhage: a meta-analysis of randomized controlled trials. Neurosurg Focus.

[13] Etminan N, Macdonald RL (2017). Management of aneurysmal subarachnoid hemorrhage. Handb Clin Neurol.

[14] Brami J, Chousterman B, Boulouis G, Dorze ML, Majlath M, Saint-Maurice JP (2020). Delayed cerebral infarction is systematically associated with a cerebral vasospasm of large intracranial arteries. Neurosurgery.

[15] Stienen MN, Smoll NR, Weisshaupt R, Fandino J, Hildebrandt G, Studerus-Germann A (2014). Delayed cerebral ischemia predicts neurocognitive impairment following aneurysmal subarachnoid hemorrhage. World Neurosurg.

[16] Steiner T, Juvela S, Unterberg A, Jung C, Forsting M, Rinkel G (2013). European stroke organization guidelines for the management of intracranial aneurysms and subarachnoid haemorrhage. Cerebrovasc Dis.

[17] Allen GS, Ahn HS, Preziosi TJ, Battye R, Boone SC, Boone SC (1983). Cerebral arterial spasm--a controlled trial of nimodipine in patients with subarachnoid hemorrhage. N Engl J Med.

[18] Petruk KC, West M, Mohr G, Weir BK, Benoit BG, Gentili F (1988). Nimodipine treatment in poor-grade aneurysm patients. Results of a multicenter double-blind placebo-controlled trial. J Neurosurg.

[19] Pickard JD, Murray GD, Illingworth R, Shaw MD, Teasdale GM, Foy PM (1989). Effect of oral nimodipine on cerebral infarction and outcome after subarachnoid haemorrhage: British aneurysm nimodipine trial. BMJ.

[20] Hayashi K, Hirao T, Sakai N, Nagata I, group J-Ns (2014). Current status of endovascular treatment for vasospasm following subarachnoid hemorrhage: analysis of JR-NET2. Neurol Med Chir (Tokyo).

[21] Fuwa I, Mayberg M, Gadjusek C, Harada T, Luo Z (1993). Enhanced secretion of endothelin by endothelial cells in response to hemoglobin. Neurol Med Chir (Tokyo).

[22] Lin G, Macdonald RL, Marton LS, Kowalczuk A, Solenski NJ, Weir BK (2001). Hemoglobin increases endothelin-1 in endothelial cells by decreasing nitric oxide. Biochem Biophys Res Commun.

[23] Kohno M, Yasunari K, Yokokawa K, Murakawa K, Horio T, Kanayama Y (1990). Thrombin stimulates the production of immunoreactive endothelin-1 in cultured human umbilical vein endothelial cells. Metabolism.

[24] Yanagisawa M, Kurihara H, Kimura S, Tomobe Y, Kobayashi M, Mitsui Y (1988). A novel potent vasoconstrictor peptide produced by vascular endothelial cells. Nature.

[25] Seifert V, Loffler BM, Zimmermann M, Roux S, Stolke D (1995). Endothelin concentrations in patients with aneurysmal subarachnoid hemorrhage. Correlation with cerebral vasospasm, delayed ischemic neurological deficits, and volume of hematoma. J Neurosurg.

[26] Davenport AP, Hyndman KA, Dhaun N, Southan C, Kohan DE, Pollock JS (2016). Endothelin. Pharmacol Rev.

[27] Roux S, Breu V, Giller T, Neidhart W, Ramuz H, Coassolo P (1997). Ro 61-1790, a new hydrosoluble endothelin antagonist: general pharmacology and effects on experimental cerebral vasospasm. J Pharmacol Exp Ther.

[28] Vajkoczy P, Meyer B, Weidauer S, Raabe A, Thome C, Ringel F (2005). Clazosentan (AXV-034343), a selective endothelin a receptor antagonist, in the prevention of cerebral vasospasm following severe aneurysmal subarachnoid hemorrhage: results of a randomized, double-blind, placebo-controlled, multicenter phase IIa study. J Neurosurg.

[29] Macdonald RL, Kassell NF, Mayer S, Ruefenacht D, Schmiedek P, Weidauer S (2008). Clazosentan to overcome neurological ischemia and infarction occurring after subarachnoid hemorrhage (CONSCIOUS-1): randomized, double-blind, placebo-controlled phase 2 dose-finding trial. Stroke.

[30] Fujimura M, Joo JY, Kim JS, Hatta M, Yokoyama Y, Tominaga T (2017). Preventive effect of clazosentan against cerebral vasospasm after clipping surgery for aneurysmal subarachnoid hemorrhage in Japanese and Korean patients. Cerebrovasc Dis.

[31] Higashida RT, Bruder N, Gupta R, Guzman R, Hmissi A, Marr A (2019). Reversal of vasospasm with clazosentan after aneurysmal subarachnoid hemorrhage: a pilot study. World Neurosurg.

[32] Macdonald RL, Higashida RT, Keller E, Mayer SA, Molyneux A, Raabe A (2011). Clazosentan, an endothelin receptor antagonist, in patients with aneurysmal subarachnoid haemorrhage undergoing surgical clipping: a randomised, double-blind, placebo-controlled phase 3 trial (CONSCIOUS-2). Lancet Neurol.

[33] Macdonald RL, Higashida RT, Keller E, Mayer SA, Molyneux A, Raabe A (2012). Randomized trial of clazosentan in patients with aneurysmal subarachnoid hemorrhage undergoing endovascular coiling. Stroke.

[34] Endo H, Hagihara Y, Kimura N, Takizawa K, Niizuma K, Togo O, et al. Effects of clazosentan on cerebral vasospasm-related morbidity and all-cause mortality after aneurysmal subarachnoid hemorrhage: two randomized phase 3 trials in Japanese patients. J Neurosurg. 2022;137:1–11. 10.3171/2022.2.JNS212914 35364589

[35] Mayer SA, Aldrich EF, Bruder N, Hmissi A, Macdonald RL, Viarasilpa T (2019). Thick and diffuse subarachnoid blood as a treatment effect modifier of Clazosentan after subarachnoid hemorrhage. Stroke.

[36] Aldrich EF, Higashida R, Hmissi A, Le EJ, Macdonald RL, Marr A, et al. Thick and diffuse cisternal clot independently predicts vasospasm-related morbidity and poor outcome after aneurysmal subarachnoid hemorrhage. J Neurosurg. 2020;134:1–9. 10.3171/2020.3.JNS193400 32442971

[37] Jaja BN, Cusimano MD, Etminan N, Hanggi D, Hasan D, Ilodigwe D (2013). Clinical prediction models for aneurysmal subarachnoid hemorrhage: a systematic review. Neurocrit Care.

[38] Wilson JT, Hareendran A, Grant M, Baird T, Schulz UG, Muir KW (2002). Improving the assessment of outcomes in stroke: use of a structured interview to assign grades on the modified Rankin scale. Stroke.

[39] Wilson JT, Pettigrew LE, Teasdale GM (1998). Structured interviews for the Glasgow outcome scale and the extended Glasgow outcome scale: guidelines for their use. J Neurotrauma.

[40] Chong ZZ (2016). S100B raises the alert in subarachnoid hemorrhage. Rev Neurosci.

[41] Teasdale G, Murray G, Parker L, Jennett B (1979). Adding up the Glasgow coma score. Acta Neurochir Suppl (Wien).

[42] Vergouwen MD, Vermeulen M, van Gijn J, Rinkel GJ, Wijdicks EF, Muizelaar JP (2010). Definition of delayed cerebral ischemia after aneurysmal subarachnoid hemorrhage as an outcome event in clinical trials and observational studies: proposal of a multidisciplinary research group. Stroke.

[43] Abruzzo T, Moran C, Blackham KA, Eskey CJ, Lev R, Meyers P (2012). Invasive interventional management of post-hemorrhagic cerebral vasospasm in patients with aneurysmal subarachnoid hemorrhage. J Neurointerv Surg.

[44] Brott T, Adams HP, Olinger CP, Marler JR, Barsan WG, Biller J (1989). Measurements of acute cerebral infarction: a clinical examination scale. Stroke.

[45] Teasdale GM, Drake CG, Hunt W, Kassell N, Sano K, Pertuiset B (1988). A universal subarachnoid hemorrhage scale: report of a committee of the world federation of neurosurgical societies. J Neurol Neurosurg Psychiatry.

[46] Nasreddine ZS, Phillips NA, Bedirian V, Charbonneau S, Whitehead V, Collin I (2005). The Montreal cognitive assessment, MoCA: a brief screening tool for mild cognitive impairment. J Am Geriatr Soc.

[47] Stienen MN, Geisseler O, Velz J, Maldaner N, Sebok M, Dannecker N (2019). Influence of the intensive care unit environment on the reliability of the Montreal cognitive assessment. Front Neurol.

[48] Williams LS, Weinberger M, Harris LE, Clark DO, Biller J (1999). Development of a stroke-specific quality of life scale. Stroke.

[49] Boosman H, Passier PE, Visser-Meily JM, Rinkel GJ, Post MW (2010). Validation of the stroke specific quality of life scale in patients with aneurysmal subarachnoid haemorrhage. J Neurol Neurosurg Psychiatry.

[50] Morley D, Dummett S, Kelly L, Dawson J, Fitzpatrick R, Jenkinson C (2016). Validation of the Oxford participation and activities questionnaire. Patient Relat Outcome Meas.

[51] Herdman M, Gudex C, Lloyd A, Janssen M, Kind P, Parkin D (2011). Development and preliminary testing of the new five-level version of EQ-5D (EQ-5D-5L). Qual Life Res.

[52] Shen Y, Dong Z, Pan P, Shi H, Song Y (2018). Risk factors for mild cognitive impairment in patients with aneurysmal subarachnoid hemorrhage treated with endovascular coiling. World Neurosurg.

[53] Springer MV, Schmidt JM, Wartenberg KE, Frontera JA, Badjatia N, Mayer SA (2009). Predictors of global cognitive impairment 1 year after subarachnoid hemorrhage. Neurosurgery.

[54] Chalard K, Szabo V, Pavillard F, Djanikian F, Dargazanli C, Molinari N (2021). Long-term outcome in patients with aneurysmal subarachnoid hemorrhage requiring mechanical ventilation. PLoS One.

[55] Vergouwen MD, Etminan N, Ilodigwe D, Macdonald RL (2011). Lower incidence of cerebral infarction correlates with improved functional outcome after aneurysmal subarachnoid hemorrhage. J Cereb Blood Flow Metab.

[56] Kreiter KT, Mayer SA, Howard G, Knappertz V, Ilodigwe D, Sloan MA (2009). Sample size estimates for clinical trials of vasospasm in subarachnoid hemorrhage. Stroke.

[57] Roux S, Kovats D, Mayer SA (2021). Impact of infarct aetiology on lesion size and clinical outcome in high-risk patients for vasospasm with thick and diffuse aSAH (abstract).

[58] Weidauer S, Lanfermann H, Raabe A, Zanella F, Seifert V, Beck J (2007). Impairment of cerebral perfusion and infarct patterns attributable to vasospasm after aneurysmal subarachnoid hemorrhage: a prospective MRI and DSA study. Stroke.

[59] Biesbroek JM, Weaver NA, Biessels GJ (2017). Lesion location and cognitive impact of cerebral small vessel disease. Clin Sci (Lond).

[60] Schmidt JM, Wartenberg KE, Fernandez A, Claassen J, Rincon F, Ostapkovich ND (2008). Frequency and clinical impact of asymptomatic cerebral infarction due to vasospasm after subarachnoid hemorrhage. J Neurosurg.

[61] Zheng K, Zhong M, Zhao B, Chen SY, Tan XX, Li ZQ (2019). Poor-grade aneurysmal subarachnoid hemorrhage: risk factors affecting clinical outcomes in intracranial aneurysm patients in a multi-center study. Front Neurol.

[62] Suarez JI, Sheikh MK, Macdonald RL, Amin-Hanjani S, Brown RD, de Oliveira Manoel AL (2019). Common data elements for unruptured intracranial aneurysms and subarachnoid hemorrhage clinical research: a National Institute for neurological disorders and stroke and National Library of medicine project. Neurocrit Care.

[63] Inagawa T (2016). Risk factors for cerebral vasospasm following aneurysmal subarachnoid hemorrhage: a review of the literature. World Neurosurg.

