# Supplementary material for: The REACT study: design of a randomized phase 3 trial to assess the efficacy and safety of clazosentan for preventing deterioration due to delayed cerebral ischemia after aneurysmal subarachnoid hemorrhage
Source: BMC Neurol. 2022 Dec 20;22:492. doi: 10.1186/s12883-022-03002-8 (PMC9763815; doi:10.1186/s12883-022-03002-8)
Supplement: Supplementary file 1 — Additional file 1. Trial registration data. [file 12883_2022_3002_MOESM1_ESM.docx]

The REACT study: Design of a randomized phase 3 trial to assess the efficacy and safety of clazosentan for preventing deterioration due to delayed cerebral ischemia after aneurysmal subarachnoid hemorrhage

Trial registration data

| **Data category** | **Information*** |
| --- | --- |
| Primary registry and trial identifying number | ClinicalTrials.gov NCT03585270 |
| Date of registration in primary registry | July 12, 2018 |
| Secondary identifying numbers | EudraCT Number: 2018-000241-39 |
| Source(s) of monetary or material support | Idorsia Pharmaceuticals Ltd. |
| Primary sponsor | Idorsia Pharmaceuticals Ltd. |
| Contact for public queries | NB - Nicolas.BRUDER@ap-hm.fr |
| Contact for scientific queries | Idorsia Pharmaceuticals Ltd. - [clinical-trials-disclosure@idorsia.com](mailto:clinical-trials-disclosure@idorsia.com) |
| Public title | Clinical research study with clazosentan to evaluate its effects on preventing complications due to the narrowing of the blood vessels (vasospasm) in the brain, caused by bleeding onto the surface of the brain. |
| Scientific title | REACT: pRevention and trEatment of vAsospasm with ClazosentTan - A prospective, multi-center, double-blind, randomized, placebo-controlled, parallel‑group, phase 3 study to assess the efficacy and safety of clazosentan in preventing clinical deterioration due to delayed cerebral ischemia (DCI), in adult subjects with aneurysmal subarachnoid hemorrhage (aSAH) |
| Countries of recruitment | United States, Austria, Belgium, Canada, Czechia, Denmark, Finland, France, Germany, Hungary, Israel, Italy, Poland, Spain, Sweden |
| Health condition(s) or problem(s) studied | Aneurysmal subarachnoid hemorrhage |
| Intervention(s) | Active comparator: clazosentan continuous intravenous infusion (15 mg/hour) for up to 14 days |
|  | Placebo comparator at the same infusion rate as clazosentan for up to 14 days |
| Key inclusion and exclusion criteria | Ages eligible for study: 18 years to 70 years  Sexes eligible for study: all  Accepts healthy volunteers: no  Inclusion/exclusion criteria: see Table 1 and 2 |
| Study type | Interventional |
|  | Allocation: randomized; Intervention model: parallel assignment; Masking: double blind |
|  | Primary purpose: prevention |
|  | Phase 3 |
| Date of first enrolment | December 15, 2018 |
| Target sample size | 400 |
| Recruitment status | Recruiting |
| Primary outcome | Occurrence of clinical deterioration due to delayed cerebral ischemia (DCI) from study drug initiation up to 14 days post-study drug initiation |
| Secondary outcomes | 1. Occurrence of clinically relevant cerebral infarction at Day 16 post-study drug initiation  A clinical relevant cerebral infarction is defined as: all-cause cerebral infraction ≥ 5 cm^3^ or cerebral infarction < 5 cm^3^ in subjects with clinical deterioration due to delayed cerebral ischemia (DCI).  Cerebral infarction refers to new or worsened infarcts and is determined by central radiology review comparing the total volume of infarcts on the computed tomography (CT) scan performed 16 days after study drug initiation with the CT performed just prior to randomization.  2. Long-term clinical outcome assessed by the modified Rankin Scale (mRS) at Week 12 post-aSAH, dichotomized into poor outcome (score ≥ 3) and good outcome (score < 3)  3. Long-term clinical outcome assessed by the Glasgow Outcome Scale Extended (GOSE) at Week 12 post-aSAH, dichotomized as follows: poor outcome (score ≤ 4) and good outcome (score > 4) |

*Idorsia Pharmaceuticals Ltd. Clinical research study with clazosentan to evaluate its effects on preventing complications due to the narrowing of the blood vessels (vasospasm) in the brain, caused by bleeding onto the surface of the brain (REACT). Available from: <https://clinicaltrials.gov/ct2/show/record/NCT03585270>.

Idorsia Pharmaceuticals Ltd. A prospective, multi-center, double-blind, randomized, placebo-controlled, parallel-group, Phase 3 study to assess the efficacy and safety of clazosentan in preventing clinical deterioration due to delayed cerebral ischemia (DCI), in adult subjects with aneurysmal subarachnoid hemorrhage (aSAH). Available from: <https://www.clinicaltrialsregister.eu/ctr-search/search?query=2018-000241-39>.
