# Supplementary material for: The REACT study: design of a randomized phase 3 trial to assess the efficacy and safety of clazosentan for preventing deterioration due to delayed cerebral ischemia after aneurysmal subarachnoid hemorrhage
Source: BMC Neurol. 2022 Dec 20;22:492. doi: 10.1186/s12883-022-03002-8 (PMC9763815; doi:10.1186/s12883-022-03002-8)

The REACT study: Design of a randomized phase 3 trial to assess the efficacy and safety of clazosentan for preventing deterioration due to delayed cerebral ischemia after aneurysmal subarachnoid hemorrhage

Definition of “thick and diffuse clot” on hospital admission CT scan

A “thick and diffuse clot” is defined as a thick confluent clot, more than 4 mm in thickness, involving 3 or more basal cisterns.

A basal cistern contains a thick clot, if SAH completely fills the cistern or a part of a larger cistern (e.g., Sylvian fissure) and is at least 4 mm in thickness measured along the shortest dimension. Care should be taken to not assess the clot thickness where the cistern is primarily in the axial plane as it will artificially increase the apparent thickness of the clot. A single isolated spot with 4 mm thick SAH is not sufficient to designate a cistern as “thick clot”. The clot should occupy a significant portion of the cistern. At least 3 cisterns must contain a thick clot.

The following guidelines should be used to confirm that the subarachnoid blood in a basal cistern is thick:

- SAH maintains a thickness of 4 mm or more over at least a 20 mm extent of the cistern.
- The cistern appears expanded because of SAH.
- There is a “negative” contrast with hyper-dense subarachnoid blood outlining subarachnoid structures.

In order to determine the overall extent of the hemorrhage, the following basal cisterns (see below figure) must be assessed at different levels (i.e., all contiguous slices from the inferior aspect of the brain stem to the most superior aspect of the Sylvian fissures), and at least 3 of the cisterns must contain a thick clot in order to be eligible for the study.

- left Sylvian fissure (green)
- right Sylvian fissure (green)
- supra sellar cistern (red)
- perimesencephalic cistern (yellow)
- anterior inter-hemispheric fissure (blue)
- prepontin cistern (not marked on the figure below)


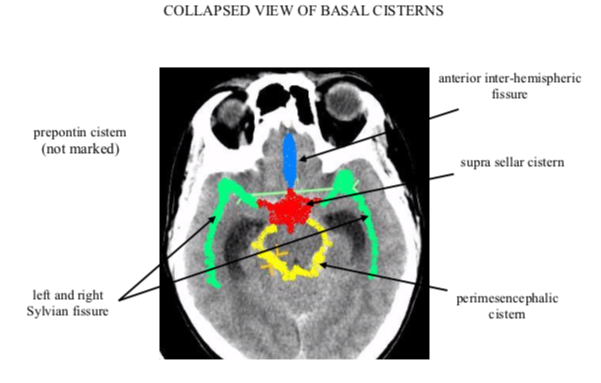

Supplement: Supplementary file 2 — Additional file 2. Definition of “thick and diffuse clot” on hospital admission CT scan. [file 12883_2022_3002_MOESM2_ESM.docx]
