# Supplementary material for: The REACT study: design of a randomized phase 3 trial to assess the efficacy and safety of clazosentan for preventing deterioration due to delayed cerebral ischemia after aneurysmal subarachnoid hemorrhage
Source: BMC Neurol. 2022 Dec 20;22:492. doi: 10.1186/s12883-022-03002-8 (PMC9763815; doi:10.1186/s12883-022-03002-8)
Supplement: Supplementary file 3 — Additional file 3. Changes to the protocol occurring after study start. [file 12883_2022_3002_MOESM3_ESM.docx]

The REACT study: Design of a randomized phase 3 trial to assess the efficacy and safety of clazosentan for preventing deterioration due to delayed cerebral ischemia after aneurysmal subarachnoid hemorrhage

Changes to the protocol occurring after study start

Protocol Version 7 - Issue date: 18 February 2022

| 7 January 2020 | Substantial amendment to add a Quality of Life assessment at 24 weeks (6 months) post-aSAH.   - The EQ-5D questionnaire was added at Week 24 post-aSAH. - The End‑of‑Study (EOS) visit at the individual patient level has been rescheduled to Week 24 post‑aSAH and the previous EOS visit has been renamed ‘Week 12 visit’. - Serious adverse event reporting was extended from up to 3 months to up to 6 months. - Clarification on supportive data collection for the primary endpoint has been provided. - The rules for rescue therapy usage have been clarified. |
| --- | --- |
| 2 July 2020 | Substantial amendment to describe the follow-up and collection of data until Day 14 post-study drug initiation for patients who are discharged from the study site prior to Day 14.   - A follow-up visit/phone call was introduced for patients who were discharged from the study site prior to Day 14 post-study drug initiation. The data to be collected and recorded during this follow-up were described. - It was explained how patients who were discharged prior to Day 14 could meet the primary efficacy endpoint based on data collected between discharge and Day 14. - A separate dedicated section was added to describe the observation period for the primary endpoint. - Information concerning image archiving at the study sites was added. |
| 29 April 2021 | Substantial amendment to discontinue recruitment into the Early Treatment (ET) group following a recommendation received by the study Independent Data Monitoring Committee on 2 April 2021. The ET group included those patients who had already developed vasospasm with no significant neurological deterioration.   - The decision to discontinue the recruitment into the ET group was not based on a planned interim efficacy analysis, nor on urgent safety observations but a low rate of recruitment into this cohort since the outset of the study, making the contribution of these patients to the overall study futile. - In a supplementary analysis, the primary efficacy analysis is repeated including the patient population (ET versus non-ET treatment) as an additional adjustment factor. |
| 18 February 2022 | Substantial amendment to modify the definition of the main secondary efficacy endpoint and the hierarchical statistical testing strategy related to the other secondary endpoints.   - The main secondary endpoint definition was updated: in addition to the already existing all-cause infarcts ≥ 5 cm^3^ at Day 16 post-study drug initiation, clinically relevant infarcts < 5 cm^3^ have been added. The latter are defined as those new or worsened infarcts < 5 cm^3^ that occur in patients with clinical deterioration due to delayed cerebral ischemia as adjudicated by the Clinical Event Committee. - Routine blinded monitoring of the event rate during the REACT study revealed a lower‑than‑expected incidence of infarcts ≥ 5 cm^3^ resulting in insufficient power to detect a treatment effect. This led to an expansion of the endpoint definition to include smaller but clinically relevant infarcts. - This amendment includes the initial definition of the main secondary endpoint, i.e., all‑cause new or worsened infarcts ≥ 5 cm^3^ at Day 16 post-study drug initiation, as an exploratory endpoint. - This revised definition of the main secondary endpoint has no impact on the evaluation of the primary efficacy endpoint. - In addition, the modified Rankin Scale has been formally included in the statistical hierarchical testing strategy, just before the Glasgow Outcome Scale-Extended. |
