# Supplementary material for: The REACT study: design of a randomized phase 3 trial to assess the efficacy and safety of clazosentan for preventing deterioration due to delayed cerebral ischemia after aneurysmal subarachnoid hemorrhage
Source: BMC Neurol. 2022 Dec 20;22:492. doi: 10.1186/s12883-022-03002-8 (PMC9763815; doi:10.1186/s12883-022-03002-8)
Supplement: Supplementary file 5 — Additional file 5. Patient Management Guidelines. [file 12883_2022_3002_MOESM5_ESM.docx]

The REACT study: Design of a randomized phase 3 trial to assess the efficacy and safety of clazosentan for preventing deterioration due to delayed cerebral ischemia after aneurysmal subarachnoid hemorrhage

Patient Management Guidelines

It is expected that, wherever possible, the rules in the guidelines are followed. However, during the treatment period, individual patient characteristics and specific clinical situations may not always allow the strict adherence to these guidelines. Any deviation is to be documented in the patient’s source notes along with medical justification. Key measures of compliance with the Patient Management Guidelines are checked by the sponsor or representative.

**1. Main objectives**

- To reduce the incidence of predictable and preventable adverse events (AEs) via information and proactive management.
- To increase consistency and standardization in the level of care across all study sites for certain key clinical parameters.

**2. Background and rationale**

The safety profile of clazosentan has been well characterized based on the data accumulated from previously conducted Phase 2 and 3 clinical trials in which over 1500 patients with aSAH were treated with active drug, including over 300 patients at the currently tested 15 mg/hour dose. Certain AEs are associated with the administration of clazosentan, notably hypotension, lung complications (in particular pulmonary edema, pleural effusion, and respiratory failure) and anemia.

When objective blood pressure (BP) measurements were analyzed, the magnitude of the BP decrease was seen to be modest, in the order of 10%. Clazosentan has a systemic vasodilatory effect, common to endothelin receptor antagonists (ERAs).

Anemia is a class effect of ERAs, is believed to be related to hemodilution, and is typically reversible after discontinuation of clazosentan.

Fluid retention is also a common finding with ERAs. An association between a positive cumulative fluid balance and the occurrence of lung complications has been observed previously in aSAH patients, suggesting an important role of fluid management in the occurrence of these events.

The above-described events are either preventable or manageable in a typical intensive care unit (ICU) setting. Patient Management Guidelines that emphasize the maintenance of euvolemia and target BP goals, are expected to decrease the incidence of lung complications related to fluid retention and hypotension and to facilitate their management in the event they should occur.

Study drug must therefore be administered in parallel to vasopressors and fluids, as needed, and must not be initiated until BP is adequately controlled according to the below guidelines. Similarly, vasopressor and fluid therapy should not be discontinued until after the discontinuation of study drug.

During the study treatment period, all patients must be in an ICU (or equivalent environment where all protocol assessments can be performed, and these Patient Management Guidelines followed) with strict BP monitoring.

Prior to enrolling a patient into the study, BP and fluid status must be controlled, as needed, with intravenous (i.v.) vasopressors and fluid administration according to these guidelines. Conditions contraindicating increased doses of vasopressors are described in these guidelines (see General BP Control) and if present at the time of enrollment, investigator judgment is required regarding the suitability of the patient for the study.

**3. Fluid management**

***3.1 General fluid monitoring and control***

The goal of the following recommendations is to emphasize euvolemia to avoid fluid overload.

Central venous monitoring is highly recommended in all patients, for enabling administration of vasopressors, fluids, and central venous pressure (CVP) measurement.

Maintenance of i.v. fluid administration

- Administer saline, Ringer’s Lactate, or a balanced crystalloid solution (e.g., Isofundine^®^, Plasma-Lyte^®^, Stereofundin^®^) starting at 1.0 to 1.5 mL/kg/h. This hourly infusion rate should include the volume of fluid administered with the study drug and be adjusted based on other i.v. administered products.
- Evaluate for the presence or absence of euvolemia. Indicators of **euvolemia** include:

1. Total 24-hour fluid output is well matched to fluid input (i.e., within 500 mL/day)

2. Inferior vena cava (IVC) is non-collapsible with spontaneous respirations (< 40% fluctuation in diameter as observed on echocardiography) off the ventilator circuit

3. A 15% or less increase in IVC diameter (as observed on echocardiography) with inspiration in mechanically ventilated patients given a tidal volume of 10 mL/kg with a positive end expiratory pressure (PEEP) of 0 cm H_2_0

4. Stroke volume variability (SVV) is < 10% (using Cheetah NICOM^®^, Edwards FloTrac^®^, PiCCO^®^, or another advanced circulatory monitoring device)

5. Increase in stroke volume to passive leg raising is < 10% (measured with a hemodynamic monitoring device)


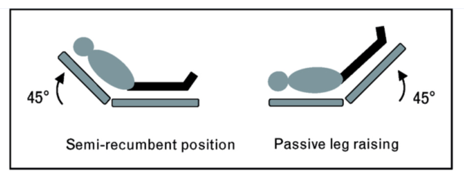


The passive leg-raising test consists of measuring the hemodynamic effects of a leg elevation up to 45°. A simple way to perform the postural maneuver is to transfer the patient from the semi-recumbent posture to the passive leg-raising position by using the automatic motion of the bed.

Conversely, the potential for **hypovolemia** is assessed by **fluid-responsiveness indicators** which are the following:

1. IVC collapses > 40% with respiratory cycle off ventilator circuit

2. CVP ≤ 5 mmHg off ventilator circuit

3. SVV is > 10% with respiratory cycle (measured with Cheetah NICOM^®^, Edwards FloTrac^®^, PiCCO^®^, or a similar hemodynamic monitoring device)

4. Passive leg raising to 45° results in a > 10% increase in left ventricular stroke volume (measured with a hemodynamic monitoring device), a > 10% increase in arterial pulse pressure (measured via arterial line), or a 5% increase in ETCO_2_ (end-tidal CO_2_).

Of note, a positive fluid responsiveness does not necessarily mean extra fluids are required. The absence of fluid responsiveness usually indicates that extra fluids are not required.

Administer a crystalloid or colloid fluid bolus if, and only if, there is evidence of potential fluid-responsive hypotension.

Warning: Administration of large fluid boluses (≥ 1 liter cumulatively over 24 hours) to patients not documented to be fluid-responsive must be avoided. The reason for administering fluid boluses with a cumulative volume of > 1 liter over 24 hours or any total 24-hour fluid input of > 4 liters (or > 60 mL/kg/day, if greater) must be documented in the source notes. For patients with a urine catheter, a positive fluid balance of more than 1000 mL over at least two consecutive days is to be justified in the patient’s medical chart.

***3.2 Permanent study drug discontinuation due to fluid retention***

Due to the potential for clazosentan to induce fluid retention, the occurrence of generalized brain edema as well as severe pulmonary edema should result in the permanent discontinuation of study drug.

**4. Blood pressure management**

***4.1 General BP control***

The goal of the following recommendations is to optimize BP by the preferential administration of vasopressors to avoid excessive administration of fluids. Fluid administration should not exceed that which is described in the Fluid Management section of these guidelines*.*

1. BP measurements obtained in the supine position by arterial line or noninvasive cuff (sphygmomanometer) are both acceptable. However, the same modality should be used consistently for each BP measurement for a given patient.

2. In the absence of vasospasm or in the presence of asymptomatic vasospasm (i.e., angiographic vasospasm without obvious clinical symptoms of vasospasm), the minimum target systolic blood pressure (SBP) should be 120 mmHg or higher. This guideline applies for most of the cases. However, it is acknowledged that in some instances, the patient’s known normal BP may be lower than this. If this is the case, then the target SBP should be the patient’s usual SBP.

3. In the presence of symptomatic cerebral vasospasm, the minimum target SBP is 150 mmHg, or higher as needed, with the target SBP titrated against clinical response. If the target SBP cannot be met because the patient’s baseline (normal) BP is low, then the target SBP should be a 30% increase from the baseline SBP.

If the target SBP is not already achieved spontaneously, BP should be raised by administering vasopressors unless specifically contraindicated. If BP is below the defined targets with more than 10% for more than 3 consecutive hours, the underlying reason is to be documented in the patient’s source notes.

Contraindications to increasing the dose of vasopressors include heart failure (due to pre-existing cardiac disease or myocardial injury in the context of aSAH) in which case the

increase in BP should be balanced against the decrease in cardiac output due to increased cardiac afterload, significant cardiac arrhythmias, pulmonary edema, and clinical or biological evidence of hypoperfusion (e.g., increase in serum lactate and creatinine).

The dosing of oral or i.v. nimodipine (if applicable) should be adjusted according to local standard practice if the above target BPs cannot be met or maintained adequately. Consider temporarily interrupting or permanently discontinuing nimodipine as appropriate.

If the target BP is already achieved spontaneously without requiring medical intervention, the potential need for vasopressors should be anticipated and the appropriate measures put into place to ensure their rapid initiation should the need arise.

***4.2 Hypotension management***

Automatic administration of bolus i.v. fluids in this scenario is discouraged unless there is compelling evidence that the patient is *fluid responsive*.

Bedside clinical assessment should occur, and intervention should be implemented in the following order:

1. Ensure that lines administering vasopressors are patent and flowing.

2. Adjust the dose of nimodipine (if applicable) and titrate the dose of vasopressor(s) upward until maximum dose is achieved.

3. If SBP does not reach the target pressure despite maximal vasopressor therapy and adjustment/discontinuation of nimodipine, study drug should be discontinued temporarily. Study drug may be restarted when the SBP stabilizes above the target. If the hypotension is refractory and suspected to be related to study drug, the study drug should be permanently discontinued.

4. Recommended vasopressors: phenylephrine or norepinephrine (noradrenaline). Epinephrine (adrenaline) and dopamine use is discouraged (due to the unpredictable effect on BP with dopamine and its potential to cause severe tachycardia, and the potential for severe arrhythmias with epinephrine).

5. In the event that the SBP goals are not reached or maintained, **“**fluid responsiveness” should be checked (see above under fluid management section).

**5. Mechanical ventilation considerations**

With low response to increased O_2_ administration and/or PEEP, be aware of the possibility of pulmonary ventilation/perfusion ratio mismatch:

- If suspicion of this phenomenon is present, standard approaches to countering hypoxia should be applied. However, PEEP and FiO_2_ should not be continuously increased beyond the usual ranges.
- If the condition does not improve, nimodipine should be stopped to check for signs of improvement. If the condition improves, then nimodipine should be discontinued permanently. If no improvement is seen after a couple of hours, it is left to the appreciation of the investigator to restart nimodipine or not. Study drug should then be temporarily interrupted to check for any potential relationship. If the condition is suspected to be related to study drug, then study drug should be permanently discontinued.

**6. General patient management**

Continuous ECG monitoring in the ICU is mandatory for the period of study drug administration. It is expected that each site has a standard protocol in place, which is adhered to for the following:

- Fever management (i.e., temperature > 38 °C/100 °F)
- Gastro-intestinal bleeding prophylaxis (e.g., ranitidine)
- Deep vein thrombosis prophylaxis (e.g., heparin 5000 units subcutaneously q12h or enoxaparin 40 mg subcutaneously once daily)
- Serum glucose monitoring and control
