# Supplementary material for: The REACT study: design of a randomized phase 3 trial to assess the efficacy and safety of clazosentan for preventing deterioration due to delayed cerebral ischemia after aneurysmal subarachnoid hemorrhage
Source: BMC Neurol. 2022 Dec 20;22:492. doi: 10.1186/s12883-022-03002-8 (PMC9763815; doi:10.1186/s12883-022-03002-8)
Supplement: Supplementary file 6 — Additional file 6. Glasgow Coma Scale and Modified Glasgow Coma Scale. [file 12883_2022_3002_MOESM6_ESM.docx]

The REACT study: Design of a randomized phase 3 trial to assess the efficacy and safety of clazosentan for preventing deterioration due to delayed cerebral ischemia after aneurysmal subarachnoid hemorrhage

Glasgow Coma Scale and Modified Glasgow Coma Scale

**Appendix 6a. Glasgow Coma Scale (GCS)**

| **TEST** | **RESPONSES** | **VALUE** |
| --- | --- | --- |
| **Eye-opening** | No response | 1 |
| **Response (1–4)** | To pain | 2 |
|  | To voice | 3 |
|  | Spontaneously | 4 |
| **Verbal response (1–5)** | No response | 1 |
|  | Incomprehensible words | 2 |
|  | Inappropriate words | 3 |
|  | Disoriented | 4 |
|  | Oriented | 5 |
| **Motor response (1–6)** | No response | 1 |
| Left arm | Abnormal extension (decerebrate) | 2 |
| Right arm | Abnormal flexion (decorticate) | 3 |
|  | Withdrawal | 4 |
|  | Localizes | 5 |
|  | Follows commands | 6 |

The GCS describes levels of consciousness by testing eye opening, verbal, and motor response*.

*Teasdale G et al. Acta Neurochir Suppl (Wien). 1979;28:13-6.

If the motor response in the left and the right arm are not the same, then the best score out of the two scores is used to determine the total GCS score.

**Intubated patients**

Subjects who are endotracheally intubated have their verbal score extrapolated from their eye-opening and best motor score according to the following table (applicable for GCS and mGCS):

**Derivation of verbal score for intubated patients**

|  | **Eye score (1–4)** | | | |
| --- | --- | --- | --- | --- |
| **Best motor score (1–6)** | **1** | **2** | **3** | **4** |
| **1** | 1 | 1 | 1 | 2 |
| **2** | 1 | 2 | 2 | 2 |
| **3** | 2 | 2 | 3 | 3 |
| **4** | 2 | 3 | 3 | 4 |
| **5** | 3 | 3 | 4 | 4 |
| **6** | 3 | 4 | 4 | 5 |

Derived verbal score = -0.3756 + Motor Score × (0.5713) + Eye-opening Score × (0.4233)*.

*Meredith W et al. J Trauma. 1998;44:839-44.

**Sedated/paralyzed patients**

Subjects who are sedated or pharmacologically paralyzed should have their sedation/paralysis interrupted/reversed for these assessments. Assessments that are unreliable due to the influence of sedation or for other reasons are not to be recorded or used to determine patient eligibility for the study. Subjects who are still under the influence of pharmacological sedation at the time of randomization or who are, for whatever reason, not evaluable for baseline and regular daily neurological assessments are excluded from the study.

**Aphasic patients**

The verbal score for aphasic patients depends on the severity of the aphasia and what verbal response the patient is actually able to produce. If the patient cannot produce speech at all, then the verbal score is 1, for no response. If the patient can speak but is replacing the expected words with inappropriate ones, then a score of 3, for inappropriate words, may be considered. If the patient does not respond to simple verbal commands, then pantomime or gestures with the body may be required.

**Appendix 6b. Modified Glasgow Coma Scale (mGCS)**

The mGCS is performed the same way as the GCS. However, the total score is calculated as the sum of the eye-opening response, the verbal response, and the worst motor response out of the two arms tested.

The mGCS is used to detect episodes of clinical deterioration due to DCI by comparison with the reference score (initially the one obtained within 30 minutes prior to study drug initiation. If a decrease of at least 2 points in the mGCS score occurs, the assessment must be repeated hourly (±15 min) for at least the first 2 hours. Thereafter, the reference score depends on the clinical evolution of the patient. After sustained improvements or worsenings in clinical status, the new reference score is recalibrated to reflect the best mGCS score attained by the patient immediately prior to an episode of clinical deterioration.

**Sedated/paralyzed patients**

Subjects who are sedated or pharmacologically paralyzed should have their sedation/paralysis interrupted/reversed for these assessments (at least once daily). However, if this is deemed unsafe for the patient, then these assessments can be waived for as long as the sedation/paralysis must continue. It is not recommended to administer long-acting sedative agents (e.g., fentanyl by continuous infusion, diazepam, barbiturates). The mGCS must not be performed in a patient who is still under the influence of sedation/paralysis. Assessments that are unreliable due to the influence of sedation or for other reasons are not to be recorded for study purposes, however the reason for the missing assessments must be documented in the medical chart.
