# Supplementary material for: The REACT study: design of a randomized phase 3 trial to assess the efficacy and safety of clazosentan for preventing deterioration due to delayed cerebral ischemia after aneurysmal subarachnoid hemorrhage
Source: BMC Neurol. 2022 Dec 20;22:492. doi: 10.1186/s12883-022-03002-8 (PMC9763815; doi:10.1186/s12883-022-03002-8)
Supplement: Supplementary file 7 — Additional file 7. Abbreviated National Institutes of Health Stroke Scale. [file 12883_2022_3002_MOESM7_ESM.docx]

The REACT study: Design of a randomized phase 3 trial to assess the efficacy and safety of clazosentan for preventing deterioration due to delayed cerebral ischemia after aneurysmal subarachnoid hemorrhage

Abbreviated National Institutes of Health Stroke Scale

| **Motor Arm and Leg:** The limb is placed in the appropriate position: extend the arms (palms down) 90 degrees (if sitting) or 45 degrees (if supine) and the leg 30 degrees (always tested supine). Drift is scored if the arm falls before 10 seconds or the leg before 5 seconds. The aphasic patient is encouraged using urgency in the voice and pantomime but not noxious stimulation. Each limb is tested in turn, beginning with the non-paretic arm. Only in the case of amputation or joint fusion at the shoulder or hip may the score be ‘9’ and the examiner must clearly write the explanation for scoring as a ‘9’.  0 = No drift, arm holds 90 (or 45) degrees for full 10 seconds.  1 = Drift, arm holds 90 (or 45) degrees, but drifts down before full 10 seconds; does not hit bed or other support.  2 = Some effort against gravity, arm cannot get to or maintain (if placed in position) 90 (or 45) degrees, drifts down to bed, but has some effort against gravity.  3 = No effort against gravity, arm falls.  4 = No movement  9 = Amputation, joint fusion explain: ______________________  **Left Arm: ________**  **Right Arm: _________**  0 = No drift, leg holds 30 degrees position for full 5 seconds.  1 = Drift, leg falls by the end of the 5 second period but does not hit bed.  2 = Some effort against gravity; leg falls to bed by 5 seconds, but has some effort against gravity.  3 = No effort against gravity, leg falls to bed immediately.  4 = No movement  9 = Amputation, joint fusion explain:_________________  **Left Leg: _________**  **Right Leg: _________** |
| --- |

The Abbreviated National Institutes of Health Stroke Scale (aNIHSS) is a measure of limb movement and strength and it only includes the motor section of the full NIHSS, which is a tool used to objectively quantify the impairment caused by a stroke*.

* Brott T et al. Stroke. 1989;20:864-70.

The aNIHSS is used to detect episodes of clinical deterioration due to DCI by comparison with the reference score (initially the one obtained within 30 minutes prior to study drug initiation). If an increase of at least 2 points in the aNIHSS score occurs, the assessment must be repeated hourly (±15 min) for at least the first 2 hours. Thereafter, the reference score depends on the clinical evolution of the patient. After sustained improvements or worsenings in clinical status, the new reference score is recalibrated to reflect the best aNIHSS score attained by the patient immediately prior to an episode of clinical deterioration.

**Sedated/paralyzed patients**

Subjects who are sedated or pharmacologically paralyzed should have their sedation/paralysis interrupted/reversed for these assessments (at least once daily). However, if this is deemed unsafe for the patient, then these assessments can be waived for as long as the sedation/paralysis must continue. It is not recommended to administer long-acting sedative agents (e.g., fentanyl by continuous infusion, diazepam, barbiturates). The aNIHSS must not be performed in a patient who is still under the influence of sedation/paralysis. Assessments that are unreliable due to the influence of sedation or for other reasons are not to be recorded for study purposes, however the reason for the missing assessments must be documented in the medical chart.
