# Supplementary material for: The REACT study: design of a randomized phase 3 trial to assess the efficacy and safety of clazosentan for preventing deterioration due to delayed cerebral ischemia after aneurysmal subarachnoid hemorrhage
Source: BMC Neurol. 2022 Dec 20;22:492. doi: 10.1186/s12883-022-03002-8 (PMC9763815; doi:10.1186/s12883-022-03002-8)
Supplement: Supplementary file 8 — Additional file 8. Glasgow Outcome Scale–Extended and Modified Rankin Scale. [file 12883_2022_3002_MOESM8_ESM.docx]

The REACT study: Design of a randomized phase 3 trial to assess the efficacy and safety of clazosentan for preventing deterioration due to delayed cerebral ischemia after aneurysmal subarachnoid hemorrhage

Glasgow Outcome Scale–Extended and Modified Rankin Scale

Glasgow Outcome Scale–Extended (GOSE)

| **Score** | **Description** |
| --- | --- |
| 1 | Dead |
| 2 | Vegetative State (VS) |
| 3 | Lower Severe Disability (Lower SD) |
| 4 | Upper Severe Disability (Upper SD) |
| 5 | Lower Moderate Disability (Lower MD) |
| 6 | Upper Moderate Disability (Upper MD) |
| 7 | Lower Good Recovery (Lower GR) |
| 8 | Upper Good Recovery (Upper GR) |

Modified Rankin Scale (mRS)

| **mRS Score** | **Description** |
| --- | --- |
| 0 | No symptoms at all |
| 1 | No significant disability despite symptoms; able to carry out all usual duties and activities |
| 2 | Slight disability; unable to carry out all previous activities, but able to look after own affairs without assistance |
| 3 | Moderate disability; requiring some help, but able to walk without assistance |
| 4 | Moderately severe disability; unable to walk without assistance and unable to attend to own bodily needs without assistance |
| 5 | Severe disability; bedridden, incontinent and requiring constant nursing care and attention |
| 6 | Dead |

Structured Interview for the Extended Glasgow Outcome Scale and the Modified Rankin Scale

Please mark (X) in the appropriate box. Please record responses to all questions (unless otherwise indicated in the text), including those concerning status before subarachnoid hemorrhage (SAH). See guidelines for further information.

| **1** | **CONSCIOUSNESS** |  |  |  |  |
| --- | --- | --- | --- | --- | --- |
|  |  | **Now** | | **Before SAH** | |
| 1.1 | **Is the person able to obey simple commands, or say any words?** | o Yes | o No | o Yes | o No |
|  |  |  | (R5, VS) |  |  |
|  | Anyone who shows ability to obey even simple commands, or utter any word, or communicate specifically in any other way is no longer considered to be in the vegetative state. Eye movements are not reliable evidence of meaningful responsiveness. Corroborate with nursing staff. Confirmation of VS requires full assessment as in the Royal College of Physician Guidelines. | | | | |

| **2** | **INDEPENDENCE IN THE HOME** |  |  |  |  |
| --- | --- | --- | --- | --- | --- |
|  |  | **Now** | | **Before SAH** | |
| 2.1 | **Is the assistance of another person at home essential every day for some activities of daily living?** | o Yes | o No | o Yes | o No |
|  |  | (upper SD) |  |  |  |
|  | For a ‘No’ answer they should be able to look after themselves at home for 24 hours, if necessary, though they need not actually look after themselves. Independence includes the ability to plan for and carry out the following activities: getting washed, putting on clean clothes without prompting, preparing food for themselves, dealing with callers, and handling minor domestic crises. The person should be able to carry out activities without needing prompting or reminding, and should be capable of being left alone overnight. | | | | |
|  |  | **Now** | | **Before SAH** | |
| 2.2 | **Do they need frequent help or someone to be around at home most of the time?** | o Yes | o No | o Yes | o No |
|  |  | (lower SD) |  |  |  |
|  | For a ‘No’ answer they should be able to look after themselves at home for up to 8 hours during the day, if necessary, though they need not actually look after themselves. | | | | |
|  |  | **Now** | | **Before SAH** | |
| 2.3 | **Does the person require constant care?** | o Yes | o No | o Yes | o No |
|  |  | (R5) |  |  |  |
|  | Constant care means that someone needs to be available at all times. Care may be provided by either a trained or an untrained caregiver. The patient will usually be bedridden and may be incontinent. Patients may not actually remain in bed all the time, but moving them from the bed to sitting will require major assistance. Patients will also need assistance with other activities. | | | | |

**Sections 3 & 4.** The following questions are about the need for assistance with some specific activities of daily life.

| **3** | **ASSISTANCE TO ATTEND TO BODILY NEEDS/ FOR WALKING** | | | | |
| --- | --- | --- | --- | --- | --- |
|  | Assistance includes physical assistance, verbal instruction, or supervision by another person. | | | | |
|  |  | **Now** | | **Before SAH** | |
| 3.1 | **Is assistance essential for eating?** (Eating without assistance: food and implements may be provided by others)*.* | o Yes | o No | o Yes | o No |
|  |  | (R4) |  |  |  |
| 3.2 | **Is assistance essential for using the toilet?** (Using toilet without assistance: reach toilet/commode; undress sufficiently; clean self; dress and leave). | o Yes | o No | o Yes | o No |
|  |  | (R4) |  |  |  |
| 3.3 | **Is assistance essential for routine daily hygiene?** (Routine hygiene: washing face, doing hair, cleaning teeth/fitting false teeth. Implements may be provided by others and this should not be considered assistance). | o Yes | o No | o Yes | o No |
|  |  | (R4) |  |  |  |
| 3.4 | **Is assistance essential for walking?** (Walking without assistance: Able to walk indoors around house or ward, may use an aid (e.g. stick/cane, walking frame/walker), however not requiring physical help or verbal instruction or supervision from another person. Wheelchair use is regarded as assistance). | o Yes | o No | o Yes | o No |
|  |  | (R4) |  |  |  |

| **4** | **ASSISTANCE TO LOOK AFTER OWN AFFAIRS** | | | | |
| --- | --- | --- | --- | --- | --- |
|  | Assistance includes physical assistance, or verbal instruction, or supervision by another person. | | | | |
|  |  | **Now** | | **Before SAH** | |
| 4.1 | **Is assistance essential for preparing a simple meal?** (For example, able to prepare breakfast or a snack) | o Yes | o No | o Yes | o No |
|  |  | (R3) |  |  |  |
| 4.2 | **Is assistance essential for basic household chores?** (For example, finding and putting away clothes, clearing up after a meal. Exclude chores that do not need to be done every day or that require heavy lifting, such as using a vacuum cleaner.) | o Yes | o No | o Yes | o No |
|  |  | (R3) |  |  |  |
| 4.3 | **Is assistance essential for looking after household expenses?** (For example, can manage day-to-day purchases, and handle money. Need not take responsibility for more complex financial matters) | o Yes | o No | o Yes | o No |
|  |  | (R3) |  |  |  |
| 4.4 | **Is assistance essential for local travel?** (Patients may drive or use public transport to get around. Ability to use a taxi is sufficient, provided the person can phone for it themselves and instruct the driver.) | o Yes | o No | o Yes | o No |
|  |  | (R3, upper SD) |  |  |  |
| 4.5 | **Is assistance essential for local shopping? (Local shopping: at least able to buy a single item.)** | o Yes | o No | o Yes | o No |
|  |  | (R3, upper SD) |  |  |  |

**Sections 5.** The next sets of questions are about how the patient usually spends his/her day.

| **5** | **USUAL DUTIES AND ACTIVITIES** |  |  |  |  |
| --- | --- | --- | --- | --- | --- |
| 5.1 | **Work** |  |  |  |  |
| 5.1.1 | **Before SAH, was the person working or seeking work (or studying as a student)?** (If the person was not employed or seeking work before SAH, or the person was retired then indicate ‘No’ and go to 5.2) | o Yes | | o No | |
| 5.1.2 | **Since SAH has there been a change in the person’s ability to work or study?** (Change in ability to work or study includes loss of employment or reduction in level of responsibility; change in education or problems with study). | o Yes | | o No | |
|  | *If ‘Yes’, how restricted are they?* | | | | |
|  | Reduced level of work e.g. change from full-time to part-time or change in level of responsibility. | o (R2, upper MD) | | | |
|  | Currently unable to work. | o (R2, lower MD) | | | |
| 5.1.3 | The person is not working due to a customary policy on sick leave, and it is difficult to assess whether they could work or not  (If this applies then the Work questions should not be used in the overall rating) | o Yes | | o No | |
| 5.2 | **Family responsibilities** |  |  |  |  |
| 5.2.1 | **Before SAH was the person looking after family at home?**  (If this was not a major role before SAH, indicate ‘No’ and go to 5.3) | o Yes | | o No | |
| 5.2.2 | **Since SAH has there been a change in their ability to look after family at home?** | o Yes | | o No | |
|  | *If ‘Yes’, how restricted are they?* | | | | |
|  | (a) Reduced responsibility for looking after family. | o Yes (R2, upper MD) | | | |
|  | (b) Currently unable to look after family. | o Yes (R2, lower MD) | | | |
| 5.3 | **Social & leisure activities** |  |  |  |  |
|  | (Social and leisure activities include hobbies and interests. Includes activities outside the home or at home. Activities outside the home: going to the pub/bar, restaurant, club, church, cinema, visiting friends, going for walks. Activities at home: involving ‘active’ participation including surfing the web, playing games, reading books, painting, home improvements). | | | | |
| 5.3.1 | **Before SAH did the person have regular free-time activities?**  (If the person had very restricted social & leisure activities before SAH then indicate ‘No’ and go to 5.4). | o Yes | | o No | |
| 5.3.2 | **Since SAH has there been a change in their ability to participate in these activities?** | o Yes | | o No | |
|  | *If ‘Yes’, how restricted are they?* | | | | |
|  | (a) Participate a bit less: at least half as often as before the SAH. | o (lower GR) | | | |
|  | (b) Participate much less: less than half as often. | o (R2, upper MD) | | | |
|  | (c) Unable to participate: rarely, if ever, take part. | o (R2, lower MD) | | | |
| 5.4 | **Family & Friendships** |  |  |  |  |
|  | (Problems with relationships include difficulties in relationships with people at home, loss of friendships or increase in isolation. Changes in the person may include: communication problems, quick temper, irritability, anxiety, insensitivity to others, mood swings, depression, and unreasonable behavior). | | | | |
| 5.4.1 | Since the SAH has the person had problems with relationships or become isolated? | o Yes | | o No | |
|  | *If ‘No’ go to 6.1 If ‘Yes’, what is the extent of disruption/strain?* | | | | |
|  | Occasional- less than weekly | o (lower GR) | | | |
|  | Frequent- once a week or more, but tolerable | o (R2, upper MD) | | | |
|  | Constant- daily & intolerable | o (R2, lower MD) | | | |
| 5.4.2 | **Before SAH were any similar problems present?** | o Yes | | o No | |

| **6** | **SYMPTOMS AS A RESULT OF THE SAH** |  |  |  |  |
| --- | --- | --- | --- | --- | --- |
| 6.1 | **Are there any other current problems relating to the SAH, which affect daily life?** | o Yes | | o No | |
|  | Problems may include: headaches, dizziness, tiredness, sensitivity to noise or light, slowness, memory failures, and concentration problems. | (Lower GR, R1) | |  | |
| 6.2 | **SYMPTOM CHECKLIST** | **Now** | | **Before SAH** | |
|  | (If symptoms due to SAH are present GOSE = Lower GR, mRS = R1) |  |  |  |  |
| 6.2.1 | **Does the person have difficulty reading or writing?** | o Yes | o No | o Yes | o No |
| 6.2.2 | **Does the person have difficulty speaking or finding the right word?** | o Yes | o No | o Yes | o No |
| 6.2.3 | **Does the person have problems with balance or co-ordination?** | o Yes | o No | o Yes | o No |
| 6.2.4 | **Does the person have visual problems?** | o Yes | o No | o Yes | o No |
| 6.2.5 | **Does the person have numbness (face, arms, legs, hands, feet)?** | o Yes | o No | o Yes | o No |
| 6.2.6 | **Has the person experienced loss of movement (face, arms, legs, hands, feet)?** | o Yes | o No | o Yes | o No |
| 6.2.7 | **Does the person have difficulty with swallowing?** | o Yes | o No | o Yes | o No |
| 6.2.8 | **Any other symptoms not already recorded or taken into consideration in 6.1 or 6.2?** | o Yes | o No | o Yes | o No |
|  | (Please record: ………………………………………………….…… ………………………………………………………………….….… ………………………………………………………………….….…) |  |  |  |  |

|  |  | | |  |  |  |  | |
| --- | --- | --- | --- | --- | --- | --- | --- | --- |
| *Rankin Grade =* |  |  |  |  | *GOSE Score =* |  | |  |
|  |  | | |  |  |  |  | |

Calculation of Glasgow Outcome Scale (extended) and Modified Rankin Scale scores

GOSE and mRS scores are derived by the interviewer from the responses to the individual interview questions following the rules detailed below:

1. The individual responses are examined and items on which there were existing limitations prior to the SAH are not used in calculating the overall scores i.e., if there is a ‘yes’ answer in the ‘before SAH’ column in any of the items 2.1, 2.2, 2.3, 3.1, 3.2, 3.3, 3.4, 4.1, 4.2, 4.3, 4.4, 4.5, 5.4.2, 6.2.1, 6.2.2, 6.2.3, 6.2.4, 6.2.5, 6.2.6, 6.2.7, or 6.2.8, indicating an existing problem prior to the SAH, then that particular item is discounted (not considered). In sections 5.1.1, 5.2.1, 5.3.1, if there is a ‘no’ answer for the status prior to the SAH, then that specific section is discounted. If there is a ‘yes’ response to item number 5.1.3, then section 5.1 should be discounted when calculating the overall scores.

2. Corresponding GOSE (VS to upper GR) and mRS (R5 to R0) categories are given in brackets beside specific responses in the interview. Some responses are relevant to scoring one scale but not the other.

3. The overall rating is the lowest disability category indicated by the individual answers (after discounting limitations or problems already existing prior to the SAH). VS is the lowest GOSE category and upper GR is the highest; mRS 5 is the lowest category, and mRS 0 is the highest. If the patient has no limitations or symptoms then the GOSE score is upper GR, and the mRS grade is 0.
